# Hierarchical and stage-specific regulation of murine cardiomyocyte maturation by serum response factor

**DOI:** 10.1038/s41467-018-06347-2

**Published:** 2018-09-21

**Authors:** Yuxuan Guo, Blake D. Jardin, Pingzhu Zhou, Isha Sethi, Brynn N. Akerberg, Christopher N Toepfer, Yulan Ai, Yifei Li, Qing Ma, Silvia Guatimosim, Yongwu Hu, Grigor Varuzhanyan, Nathan J. VanDusen, Donghui Zhang, David C. Chan, Guo-Cheng Yuan, Christine E. Seidman, Jonathan G. Seidman, William T. Pu

**Affiliations:** 1 Department of Cardiology, Boston Children’s Hospital, 300 Longwood Avenue, Boston, MA 02115 USA; 2 Department of Biology, Boston University, 5 Cummington Mall, Boston, MA 02215 USA; 3 Department of Biostatistics and Computational Biology, Dana-Farber Cancer Institute, 450 Brookline Avenue, Boston, MA 02215 USA; 4 Department of Genetics, Harvard Medical School, 77 Avenue Louis Pasteur, Boston, MA 02115 USA; 5 Radcliffe Department of Medicine and Wellcome Trust Centre for Human Genetics, University of Oxford, Roosevelt Drive, Oxford, OX3 7BN UK; 6 Key Laboratory of Birth Defects and Related Diseases of Women and Children of MOE, Department of Pediatrics, West China Second University Hospital, Sichuan University, 610041 Chengdu, Sichuan China; 7 Department of Physiology and Biophysics, Institute of Biological Sciences, Universidade Federal de Minas Gerais, Av. Antônio Carlos 6627, Belo Horizonte, MG CEP: 31270-901 Brazil; 8 Division of Biology and Biological Engineering, California Institute of Technology, 1200 East California Boulevard, MC 114-96, Pasadena, CA 91125 USA; 9 Division of Cardiovascular Medicine, Brigham and Women’s Hospital, 75 Francis Street, Boston, MA 02115 USA; 10 Howard Hughes Medical Institute, 4000 Jones Bridge Road, Chevy Chase, MD 20815 USA; 11 Harvard Stem Cell Institute, 7 Divinity Avenue, Cambridge, MA 02138 USA; 12 Present Address: Wenzhou Medical University, School of Life Sciences, Wenzhou, China; 13 Present Address: Hubei Collaborative Innovation Center for Green Transformation of Bio-resources, Hubei Key Laboratory of Industrial Biotechnology, College of Life Sciences, Hubei University, 430062 Wuhan, China

## Abstract

After birth, cardiomyocytes (CM) acquire numerous adaptations in order to efficiently pump blood throughout an animal’s lifespan. How this maturation process is regulated and coordinated is poorly understood. Here, we perform a CRISPR/Cas9 screen in mice and identify serum response factor (SRF) as a key regulator of CM maturation. Mosaic SRF depletion in neonatal CMs disrupts many aspects of their maturation, including sarcomere expansion, mitochondrial biogenesis, transverse-tubule formation, and cellular hypertrophy. Maintenance of maturity in adult CMs is less dependent on SRF. This stage-specific activity is associated with developmentally regulated SRF chromatin occupancy and transcriptional regulation. SRF directly activates genes that regulate sarcomere assembly and mitochondrial dynamics. Perturbation of sarcomere assembly but not mitochondrial dynamics recapitulates SRF knockout phenotypes. SRF overexpression also perturbs CM maturation. Together, these data indicate that carefully balanced SRF activity is essential to promote CM maturation through a hierarchy of cellular processes orchestrated by sarcomere assembly.

## Abstract (web summary)

The processes regulating cardiomyocyte (CM) maturation are unclear. Here, the authors show that serum response factor regulates CM maturation only in neonatal CMs through stage-specific chromatin occupancy that affects cell size, sarcomere and transverse-tubule organization, and mitochondria

## Introduction

Adult cardiomyocytes (CMs) generate forceful contractions billions of times during the lifespan of an adult human. Specialized features that adapt CMs for this unique activity include their large rod-like shape, nearly crystalline sarcomere organization, robust oxidative metabolic capacity, expression of mature sarcomere gene isoforms, exit from the cell cycle, and an extensive network of transverse tubules (T-tubules), which are plasma membrane invaginations that facilitate synchronized calcium release^1,2^. In contrast to adult CMs, these specialized features are absent or underdeveloped in fetal and neonatal CMs, which are smaller, proliferative, glycolytic cells with less organized sarcomeres, fewer and smaller mitochondria, and no T-tubules. The dramatic transition between fetal and adult phenotypes, termed CM maturation, occurs in the first few weeks following birth (approximately P0–P21) in mice. Little is known about the signals and transcriptional machineries that coordinate CM maturation. Likewise, it is unclear if the maintenance of maturity is regulated by the same or distinct mechanisms.

Understanding CM maturation is critical to answering many major questions in cardiac biology. Because CM maturation is essential to establish proper heart functions in adults, aberrations in CM maturation could result in or exacerbate cardiomyopathies. Maturation may also be disturbed by abnormal hemodynamic loads due to congenital heart malformation, which may impact outcomes in congenital heart disease patients. CM maturation is associated with the loss of CM regeneration capacity, which occurs in the first week after birth in mice^3^. CM de-maturation, often referred as CM dedifferentiation, is likely to be important for CM regeneration^3–5^. Finally, improved understanding of normal CM maturation mechanisms is required for us to better harness the therapeutic potential of stem cell-derived CMs, which is currently limited by our inability to mature them^1^.

CM maturation studies have been held back by technical challenges. Because fully mature CMs cannot be induced or maintained in in vitro cell culture systems, these approaches are not ideal to study CM maturation. Progress using in vivo models has been slow and expensive, due to the time required to produce and mate genetically modified mouse models for each candidate gene. Moreover, studies in mice with organ-wide gene modifications have been confounded by secondary effects, such as the de-maturation-like phenotypes of heart failure^6^. We recently established an adeno-associated virus (AAV)-mediated CRISPR/Cas9-based somatic mutagenesis system (CASAAV) that provided a robust platform to study CM maturation in vivo^6,7^ (Supplementary Fig. 1a). CASAAV allows quick generation of loss-of-function mutations of a given gene specifically in neonatal CMs. Importantly, through AAV titration, this method can easily generate genetic mosaics^6,8^, which allows cell-autonomous gene functions to be probed while circumventing the confounding secondary effects of organ-wide dysfunction.

In this study, we perform a CASAAV-based screen and identified serum response factor (*Srf*) as a key regulator of CM maturation. Genetic mosaic analysis of SRF depletion and overexpression show that SRF is a stage-specific, dosage-sensitive regulator of CM maturation. Furthermore, we identify a hierarchy of maturation processes, in which sarcomere maturation was required for morphological maturation but mitochondrial biogenesis was not.

## Results

### CASAAV-based screen for T-tubule maturation factors in vivo

Reasoning that CM maturation may be regulated by factors that also regulate CM differentiation, we studied nine transcriptional regulators of CM differentiation (*Gata4*, *Gata6*, *Mef2a*, *Mef2c*, *Tead1*, *Srf*, *Tbx5*, *Nkx2.5*, and *Tead1*) as candidate maturation factors (Supplementary Fig. 1b). We performed CASAAV-based mutagenesis of each factor in postnatal day 1 (P1) CMs and assessed the impact on T-tubule formation in 1-month-old hearts. In situ T-tubule imaging and quantitative analysis by AutoTT^9^, a software that objectively quantifies T-tubule contents by normalizing T-tubule patterns to cell morphology^6,9,10^, revealed that AAV directed against *Srf* was the only treatment that caused T-tubule defects (Supplementary Fig. 1c, d). Among the remaining candidates, we had previously validated effective GATA4, NKX2-5, and TEAD1 CASAAV-mediated depletion^6,7^. Here we also validated successful depletion of SRF and GATA6 by CASAAV (Supplementary Fig. 1e, f), and we confirmed the dispensable role of TBX5 in T-tubule formation using a well-established *Tbx5*-floxed allele^11^ (Supplementary Fig. 1g). Together, these data demonstrate a unique role of *Srf* in CM maturation.

### Genetic mosaic SRF depletion in CMs

**Fig. 1:**
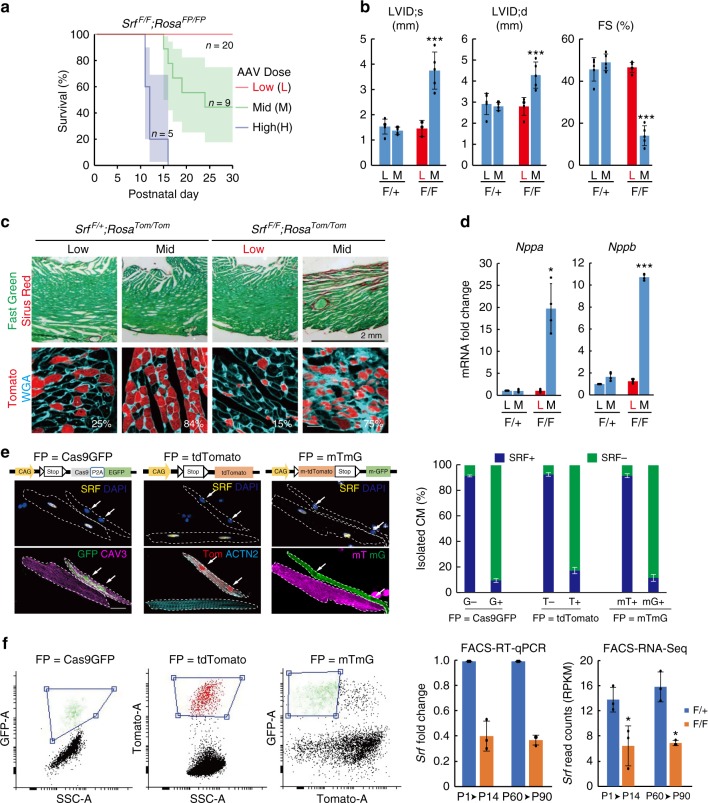
Mosaic knockout of Srf circumvents disruption of global heart function. **a** Survival curve of *Srf*^*F/F*^*;Rosa*^*FP/FP*^ mice treated with low, mid, and high doses of AAV-Cre at P1. **b** Effect of AAV-Cre dosage on heart function and chamber size at P30. Left ventricle (LV) function and size were assessed echocardiographically by measuring the LV fractional shortening (FS) and internal diameter at end systole (LVID;s) and diastole (LVID;d). *n* = 5 per group. **c** Effect of AAV-Cre doses on myocardial fibrosis. Fibrosis was measured from heart sections stained with sirus red (top) or wheat germ agglutinin (WGA, bottom). The fraction of FP+ cells in heart sections was quantified and labeled in white. **d** RT-qPCR analysis of cardiac stress marker expression from heart ventricles. *n* = 4 per group. **e** Representative images of SRF immunofluorescence in FP+ and FP− *Srf*^*F/F*^*;Rosa*^*FP/FP*^ CMs that were isolated from the same heart. Arrows point to SRF-FP+ nuclei. Quantification was shown to the right. *n* = 3 hearts. **f** Representative FACS plots and gating to sort FP+ CMs (left) and measurement of *Srf* expression in FACS-sorted FP+ CMs (right) by RT-qPCR and RNA-seq. AAV-Cre was injected at P1 and CMs analyzed at P14, or AAV-Cre was injected at P60 and CMs analyzed at P90. *n* = 3 hearts. Bar plots show mean ± SD and are overlaid by dot plots of individual data. Two-tailed Student’s *t* test: *P < 0.05, ***P < 0.001. F/+: *Srf*^*F/+*^. F/F, *Srf*^*F/F*^. Scale bar, 20 µm

Ablation of *Srf* by conventional conditional knockout (KO) technologies causes lethal dilated cardiomyopathy^12–14^, which generates secondary effects of heart stress that confound analysis of SRF functions in physiological conditions, such as CM maturation. To solve this problem, we used an AAV vector (AAV-cTNT-Cre, or AAV-Cre)^6,15^ to specifically deliver Cre recombinase into CMs in mice with well-characterized floxed *Srf* alleles (*Srf*^*F/F*^)^12^. Injection of a high (1 × 10^10^ vg g^−1^) or intermediate (5 × 10^9^ vg g^−1^) dose of AAV-Cre into P1 *Srf*^*F/F*^ mice triggered lethality and acute cardiomyopathy characterized by heart failure, ventricular dilatation, fibrosis, and the up-regulation of cardiac stress markers *Nppa* and *Nppb* (Fig. 1a–d). This is consistent with previous findings that *Srf* is essential for proper heart function^13,16,17^. We next titrated down the dose of AAV-Cre to 5 × 10^8^ vg g^−1^, which generated mosaic *Srf* inactivation in <15% CMs while maintaining normal heart morphology and function (Fig. 1a–d). This mosaic KO strategy opens the door to study the cell-autonomous role of *Srf* in CM maturation while minimizing confounding effects of heart dysfunction.

We generated *Srf*^*F/F*^*;Rosa*^*FP/FP*^ (KO) and *Srf*^*F/+*^*;Rosa*^*FP/FP*^ (control (CTRL)) mice that harbored Cre-inducible fluorescent protein (FP) reporters (either Cas9GFP, tdTomato, or mTmG, see Fig. 1e), which were essential to identify and purify Cre-activated CMs in the mosaics. Comparison of SRF immunostaining to FP expression in *Srf*^*F/F*^*;Rosa*^*FP/FP*^ mice indicated that 85–90% of FP+ CMs lacked SRF, compared to only 10% of FP− CMs (Fig. 1e). Quantitative reverse transcription-polymerase-chain reaction (RT-qPCR) and RNA-sequencing (RNA-seq) analyses of FP+ CMs that were purified by flow cytometry (fluorescence-activated cell sorting (FACS)) indicated ~60% *Srf* depletion in *Srf*^*F/F*^ CMs as compared to *Srf*^*F/+*^ CMs (Fig. 1f). This efficiency was slightly lower than the anticipated 85–90% accuracy of the FP reporters as determined by immunostaining (Fig. 1e), which likely reflects imperfect FACS performance in sorting large CMs. These data indicated that FP reporters were useful surrogate markers to identify and enrich AAV-Cre-infected CMs.

### Stage-specific role of SRF in CM maturation

**Fig. 2:**
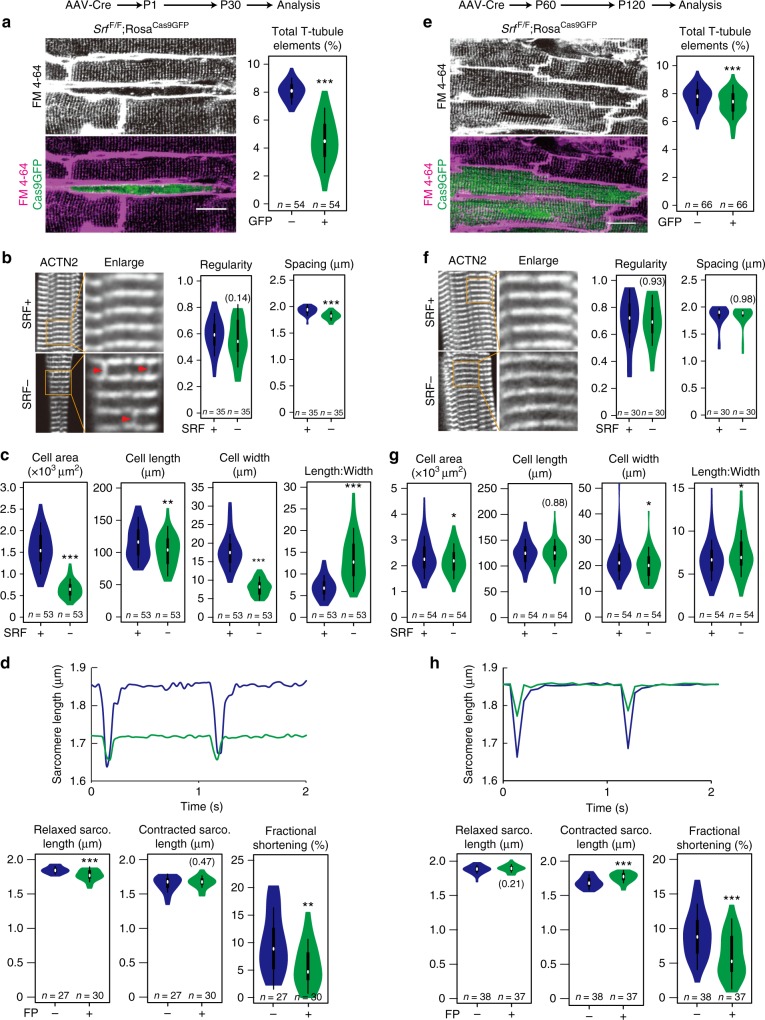
*Srf* plays a profound role in CM maturation but not the maintenance of CM maturity. **a–d** Mosaic depletion of *Srf* in neonatal CMs in vivo resulted in dramatic defects in T-tubule organization (**a**), sarcomere organization (**b**), maturational hypertrophic growth (**c**), and contraction (**d**). **e–h** *Srf* ablation in adult CMs had a minor impact on T-tubule organization (**e**), sarcomere organization (**f**), maturational hypertrophic growth (**g**), and contraction (**h**). **a**, **e** Representative in situ images of T-tubules that were labeled by membrane dye FM 4–64 and quantified by the AutoTT software^9^. Scale bar, 20 μm. **b**, **f** Confocal images of ACTN2-immunostained isolated CMs, demonstrating organization of sarcomere z-lines. Red arrowheads point to abnormal ACTN2 staining that extends between z-lines. Regularity was measured by AutoTT and Z-line spacing measured manually. **c**, **g** Quantification of the size and morphology of isolated CMs. **d**, **h** Measurement of CM contractility. Isolated CMs were paced at 1 Hz. Bright-field images were acquired and then analyzed using SarcOptiM^18^. In violin plots, white circles show the medians, box limits indicate the 25th and 75th percentiles, whiskers extend 1.5 times the interquartile range from the 25th and 75th percentiles, and polygons represent density estimates of data and extend to extreme values. Two-tailed Student’s *t* test: *P < 0.05, **P < 0.01, ***P < 0.001. Non-significant *P* values are labeled within parentheses

We first analyzed the impact of SRF depletion on key morphological hallmarks of maturation in actively maturing CMs by injecting AAV-Cre into P1 mice (neonatal KO) and analyzing 1 month later. Consistent with the CASAAV-based loss-of-function screen (Supplementary Fig. 1d, e), neonatal *Srf* KO caused dramatic T-tubule loss in FP+ *Srf*^*F/F*^ CMs as compared to FP− CTRLs (Fig. 2a). This result was further validated by immunofluorescent staining of key T-tubule markers JPH2 and CAV3 on isolated CMs (Supplementary Fig. 2a). In situ imaging revealed decreased CM size (Fig. 2a) in neonatal *Srf* KO CMs, which was confirmed by measuring CM cross-sectional area (Supplementary Fig. 2b). ACTN2, a sarcomere Z-line marker, retained a grossly normal, striated sarcomere pattern in SRF-depleted CMs, although aberrant longitudinal ACTN2 localization could be observed between some Z-lines (red arrows in Fig. 2b). The distance between Z-lines also decreased in mutant CMs (Fig. 2b). We next FACS-sorted FP+ CMs and performed electron microscopy (EM; Supplementary Fig. 2c), which confirmed grossly normal myofibrillar striations in FP+ *Srf*^*F/F*^ CMs and uncovered a significant reduction of myofibril numbers as compared to FP+ *Srf*^*F/+*^ CMs (Supplementary Fig. 2d). EM also revealed loss of the M-line, a hallmark of maturation, and the formation of bulged Z-lines (Supplementary Fig. 2e), which were consistent with the longitudinal ACTN2 staining observed in SRF-depleted CMs (Fig. 2b). Geometric analysis of isolated CMs showed that *Srf* ablation dramatically decreased projected cell area and cell width, but cell length was only slightly reduced, resulting in strikingly increased length:width ratio (Fig. 2c). Thus, hypertrophic growth of maturing CMs is blocked in the absence of SRF. Terminal deoxynucleotidyl transferase dUTP nick-end labeling (TUNEL) analyses did not label Srf KO CMs, suggesting that their severe phenotypes are not due to cell death (Supplementary Fig. 2f).

To assess the role of *Srf* in the maturation of CM physiological activity, we next assayed CM contractility and Ca^2+^ transients following neonatal SRF depletion. Bright-field live imaging of electrically paced CMs was performed to measure sarcomere contraction and relaxation^18^ (Supplementary Fig. 3a). In mutant cells, we observed dramatically lower sarcomere fractional shortening and relaxed sarcomere length (Fig. 2d), consistent with sarcomere measurements made in fixed and relaxed CMs (Fig. 2b). In contrast, contracted sarcomere length was preserved in mutant CMs (Fig. 2d). We also tested Ca^2+^ handling by recording confocal line scans of electrically paced CMs loaded with the Ca^2+^-sensitive dye Fluo-4 (when FP was tdTomato) or Rhod-2 (when FP was Cas9GFP). The mutant cells displayed reduced Ca^2+^ transient amplitude and prolonged time to peak Ca^2+^ signal (Supplementary Fig. 3b), consistent with the T-tubule defects (Fig. 2a). Together, these data show that *Srf* is essential for functional maturation of CMs, in part through its role in morphological maturation.

SRF depletion could trigger the above phenotypes through either perturbation of a specific CM maturation program or disruption of the maintenance (or homeostasis) of CM maturity. To distinguish these two mechanisms, we next injected AAV-Cre into adult (P60) animals to induce adult-specific *Srf* inactivation in CMs (adult KO). RT-qPCR and RNA-seq revealed similar *Srf* depletion efficiencies between neonatal and adult KO models (Fig. 1f). Strikingly, adult-specific depletion of SRF in CMs resulted in minor defects in T-tubule organization (Fig. 2e), sarcomere organization (Fig. 2f), and CM area, length, width, and length:width ratio (Fig. 2g). Adult KO did not change relaxed sarcomere length (Fig. 2h) or time to peak Ca^2+^ signal (Supplementary Fig. 3b) in electrically paced CMs. However, adult KO did reduce CM fractional shortening as a result of increased contracted sarcomere length (Fig. 2h), and decreased Ca^2+^ transient amplitude (Supplementary Fig. 3b). Together, these data demonstrate a profound and stage-specific role of *Srf* in CM maturation.

### Stage-specific transcriptomic regulation by SRF

**Fig. 3:**
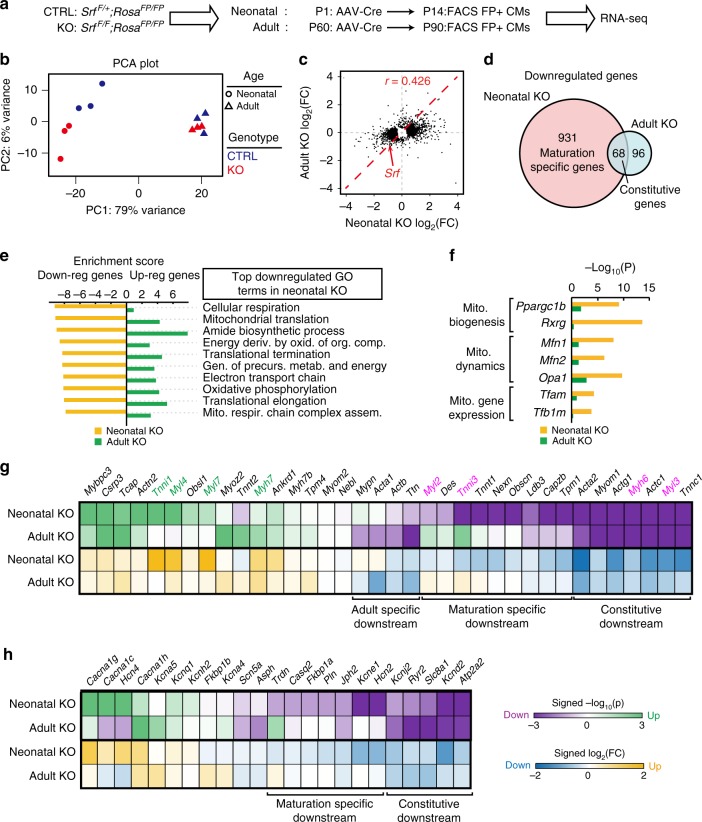
Maturation-specific transcriptional regulation by SRF. **a** Experimental design. Mosaic SRF depletion was induced at neonatal or adult stages. Transduced CMs were FACS-purified and analyzed by RNA-seq. **b** PCA plot of RNA-seq results (also see Supplementary Fig. 4a). **c** Comparison of gene expression changes in neonatal or adult *Srf* ablation. The fold changes (KO/CTRL) of differentially expressed genes (*P* value <0.05) in neonatal or adult KO models were plotted. *r*=Pearson's correlation coefficient. Red arrow points to *Srf*, which was similarly depleted in both models. **d** Venn diagram of down-regulated genes in neonatal or adult KO. **e** Gene ontology terms enriched among genes down-regulated in the neonatal KO model. The GSEA^20^ normalized enrichment scores for the top 10 terms in the neonatal KO model are shown in yellow, and the corresponding scores of the same terms for the adult KO model are shown in green. Negative and positive scores show down-regulation or up-regulation, respectively. **f** Representative mitochondrial genes that were selectively down-regulated in neonatal KO but not in adult KO. **g**, **h** Differential expression analyses of major sarcomere (**g**) and calcium handling (**h**) genes. Magenta-green and blue-yellow color scales indicate the differential expression *P* values and fold changes, respectively. Negative and positive values denote down-regulation and up-regulation, respectively. In **g**, mature (magenta gene names) and immature (green gene names) myofibrillar isoforms are highlighted. Differential expression *P* values were calculated by DESeq2^59^ with Benjamini–Hochberg correction

To determine the mechanisms by which *Srf* regulates CM maturation, we profiled transcriptome changes in both neonatal and adult SRF KO models by RNA-seq. In the neonatal KO model, AAV-Cre was delivered at P1, and CMs were analyzed at P14. In the adult KO model, AAV-Cre delivery and CM analysis occurred at P60 and P90, respectively. In both models, *Srf*^*F/F*^ (KO) and *Srf*^*F/+*^(CTRL) FP+ CMs were FACS-purified before RNA extraction; RNA-seq libraries were prepared using a protocol designed for low RNA input (Fig. 3a)^19^. Principal component analysis (PCA) showed clear separation between CTRL and KO groups in both neonatal and adult models (Supplementary Fig. 4a). We identified 999 down-regulated genes and 787 up-regulated genes in neonatal *Srf* KO (adjusted *P* value <0.05; Supplementary Fig. 4b). By contrast, using the same statistical threshold, adult *Srf* KO only caused down-regulation and up-regulation of 164 and 148 genes, respectively (Supplementary Fig. 4b). PCA better separated CTRL and KO in neonatal as compared to adult stage (Fig. 3b). The differentially expressed genes were weakly correlated (*r* = 0.426; Fig. 3c) between neonatal and adult stages. Only 6.2% of all down-regulated genes (68 genes) were down-regulated in both models (Fig. 3d). Thus, *Srf* regulates transcription in a maturation-specific manner.

We performed gene set enrichment analysis (GSEA)^20^ to identify the major biological processes downstream of *Srf*. In the neonatal KO model, the major gene ontology (GO) terms enriched among down-regulated genes were related to oxidative phosphorylation and mitochondria (Fig. 3e). Key regulators of mitochondrial biogenesis (*Ppargc1b*, *Rxrg*), mitochondria dynamics (mitofusin 1 (*Mfn1*), mitofusin 2 (*Mfn2*), *Opa1*), and mitochondrial transcription (*Tfam*, *Tfb1m*) were down-regulated upon neonatal KO (Fig. 3f). Upstream regulator analysis by Ingenuity Pathway Analysis (IPA) also identified key mitochondrial biogenesis regulators *Insr*, *Ppargc1a*, and *Esrra* (Supplementary Fig. 4c). The profound role of SRF in mitochondria and respiration in the neonatal KO model is striking, because this was not noted in previously reported models where disrupting *Srf* signaling mainly disrupts genes related to heart development and the sarcomere/actin cytoskeleton^12,14,21–24^. Indeed, heart development and muscle cell differentiation were the major down-regulated GO terms in adult *Srf* KO models (Supplementary Fig. 4d); mitochondria-related and metabolism-related genes were not enriched in adult *Srf* KO (Fig. 3e, f). These data indicate a unique stage-specific role of *Srf* in mitochondrial and metabolic maturation.

To further validate the role of *Srf* in mitochondrial maturation, we performed EM on neonatal *Srf* KO and CTRL CMs (Supplementary Fig. 5a, b). We observed a dramatic reduction of mitochondrial size and number in the *Srf* KO cells. Mitochondrial DNA, transcription, and protein components were also decreased in FACS-sorted FP+ *Srf*^*F/F*^ CMs (Supplementary Fig. 5c–e). However, in situ imaging of CMs that were labeled by tetramethylrhodamine (TMRM), a mitochondrial membrane potential indicator, was unperturbed in FP+ *Srf*^*F/F*^ CMs, suggesting retained mitochondria quality in neonatal *Srf* KO CMs (Supplementary Fig. 5f).

We next examined the expression of genes regulating sarcomere assembly and Ca^2+^ handling. Strikingly, in contrast to an expected down-regulation of most sarcomere genes^21^, we observed both up-regulation and down-regulation of sarcomere genes (Fig. 3g). Down-regulation of core sarcomere components such as *Actc1*, *Myh6*, and *Myl3* explained the sarcomere assembly defects in neonatal KO model (Fig. 2b). The up-regulation of Z-line components *Actn2*, *Tcap*, and *Csrp3* (Fig. 3g) was consistent with the excessive Z-line patterns found in neonatal KO model (Fig. 2b). These genes were also differentially expressed in the adult KO model where there was no detectable sarcomere disorganization (Fig. 2f), which suggested that the abundance of these sarcomere gene transcripts is not limiting for maintenance of sarcomere organization in adult CMs. Notably, mature myofibrillar isoforms, including *Myh6*, *Tnni3*, *Myl2* and *Myl3*, were down-regulated in neonatal *Srf* KO CMs, and the corresponding immature isoforms, *Myh7*, *Tnni1*, *Myl7*, and *Myl4*, were up-regulated (Fig. 3g, highlighted genes). This strongly suggests a key role of *Srf* in myofibrillar isoform switching, a critical transcriptional maturation hallmark^1^. We also observed down-regulation of *Atp2a2*, *Slc8a1*, *Ryr2* and up-regulation of *Cacna1c* (Fig. 3h), which likely contributed to Ca^2+^ handling defects in addition to the T-tubule phenotypes. Interestingly, we observed up-regulation of *Hcn4* (Fig. 3h), an ion channel that is specifically expressed in immature CMs^25^.

### Selective chromatin binding by SRF in maturing CMs

**Fig. 4:**
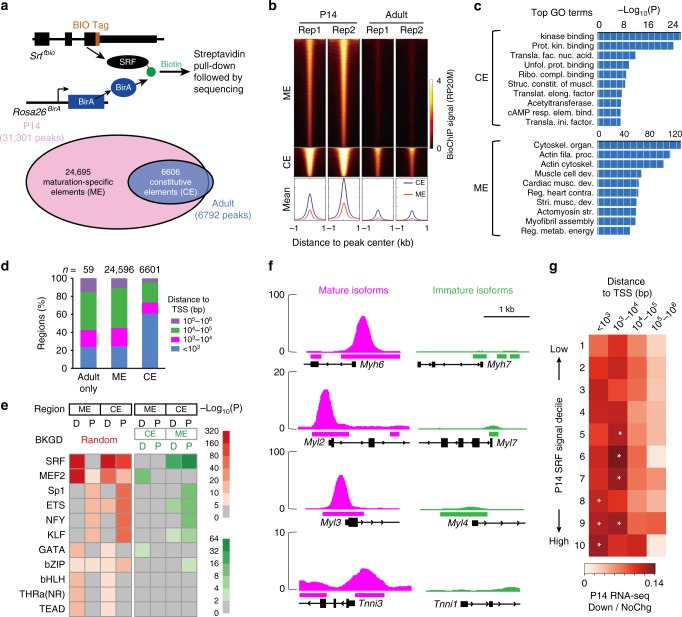
Stage-specific SRF chromatin occupancy determines its maturation-specific regulation of gene expression. **a** SRF bioChIP-Seq at P14 and adult stages identified chromatin regions bound by endogenous SRF in hearts. These regions were grouped into maturation-specific elements (MEs) uniquely present at P14, and constitutive elements (CEs) found at both P14 and adult stages, and adult-only elements. **b** SRF bioChIP-seq signal on CEs and MEs. Each row shows SRF signal of a genomic region centered on a CE or ME and extending 1 kb upstream and downstream. Plots below show average signal at these regions. **c** GO term analysis of genes associated with CEs or MEs. **d** Distance of SRF-bound regions to the nearest transcriptional start site (TSS) for peaks found at P14 only (ME), P14 and adult (CE), or adult only. **e** Analysis of TF motifs enriched in the central 200 bp of CEs and MEs at proximal (TSS ±1 kb) or distal regions. The 1000 regions with the greatest SRF signal in each group was used for this analysis. Non-redundant motifs with *P* < 10^–5^ are shown. Left and right panels show enrichment compared to randomly permuted background or the indicated set of comparison sequences, respectively. *P* values were calculated by Homer^65^ using a negative binomial distribution. **f** SRF bioChIP-seq signal at P14 at four SRF-dependent mature myofibrillar genes (magenta) and their immature paralogs (green). Called peak regions are labeled as bars below the signal plot. **g** Relationship between peak distance to TSS, peak SRF occupancy signal, and likelihood of gene down-regulation on SRF knockout. SRF-occupied regions were divided into deciles by SRF bioChIP-seq signal. Color represents the ratio of down-regulated to unchanged genes. One-tailed Fisher's exact test: *P < 0.05

In order to map SRF chromatin occupancy, we generated a knock-in allele of *Srf* that was fused to a biotin acceptor peptide (BIO tag) at the carboxyl-terminus (*Srf*^*fbio*^; Supplementary Fig. 6a). BIO is specifically biotinylated by the *Escherichia coli* biotin ligase BirA, which was expressed from the *Rosa26*^*BirA*^ allele^26^. In hearts containing both alleles, SRF was biotinylated so that it could be efficiently pulled down on immobilized streptavidin (Fig. 4a and Supplementary Fig. 6b). This system allowed us to pull-down SRF-associated chromatin in the heart in a highly sensitive and specific manner, circumventing the caveats of antibody-based chromatin immunoprecipitation^27^. We performed next-generation sequencing of SRF co-precipitated DNA (bioChIP-seq) and identified SRF binding sites in the genome. At P14 and adult stages, we obtained two biological replicates of SRF bioChIP-Seq data. There was very high correlation (*r* > 0.95) between biological repeats (Fig. 4b and Supplementary Fig. 6c, d). The SRF DNA-binding motif (the CArG motif) was the top sequence recovered by de novo motif finding (Supplementary Fig. 7a), which further validated the efficacy of this method.

BioChIP-seq identified 31,301 and 6792 high-confidence (*P* < 1 × 10^−5^) peaks at P14 and adult stages, respectively (Supplementary Fig. 6c). Among all SRF-bound regions found at P14, 24,695 (78.9%) were uniquely identified at P14 and termed maturation-specific SRF-bound elements (MEs) (Fig. 4a). By contrast, 6606 (21.1%) of P14 regions were also identified in adult CMs. These constitutive SRF-bound elements (CEs; Fig. 4a) composed 97.3% of adult SRF-bound regions. Interestingly, SRF elements with the highest occupancy signal were most likely to be retained in adult heart at CEs, with 83% of the P14 regions in the highest SRF occupancy signal decile being CEs (Fig. 4b and Supplementary Fig. 7b). However, genes regulating major heart functions such as heart development, myofibril assembly, and metabolism were more enriched neighboring MEs compared to CEs (Fig. 4c). Together, these data indicate that postnatal CM maturation is accompanied by loss of developmental SRF binding to chromatin, which likely explains SRF maturation-specific transcriptional regulation (Fig. 3).

We further characterized the properties of MEs compared to CEs. Analysis of the location of SRF sites with respect to transcriptional start sites (TSSs) showed that more than 20% of MEs and more than 60% of CEs are proximal to promoters. Considering that promoters occupy <1% of the genome, these data represent substantial enrichment of SRF occupancy near promoters for both MEs and CEs. As compared to CEs, more MEs are distal to promoters (TSS ± 1000 bp) (Fisher’s exact test: *P* < 10^−16^; Fig. 4d). We also performed motif analysis on proximal or distal CEs and MEs with the strongest SRF occupancy signal (top 1000 per group; Fig. 4e). MEs and CEs showed a similar overall motif enrichment pattern when compared to randomly permuted background. However, different sets of motifs were enriched in proximal vs. distal regions. Enriched distal co-motifs included MEF2, GATA, and TEAD, whereas enriched proximal co-motifs included MEF2, SP1, KLF, and ETS (Fig. 4e). Consistent with these data, physical interaction of SRF with GATA, TEAD, KLF, and ETS family proteins has been reported previously^28–31^. An analysis of differential motif enrichment between MEs and CEs showed that both proximal and distal CEs were more enriched for the SRF motif than MEs. Distal MEs were significantly more enriched for MEF2 and GATA motifs. Consistent with this observation, SRF binding sites in P14 hearts overlapped with 35% GATA4 binding sites and 37% MEF2A binding sites that were previously identified by ChIP-seq in the HL1 cardiac muscle cell line^27^ (Supplementary Fig. 7c). These findings imply that SRF collaboration with MEF2 and GATA family members is a potential mechanism that regulates CM maturation.

Next, we evaluated the relationship between P14 and adult chromatin occupancy and differential gene expression. Genes down-regulated in neonatal but not adult SRF KO were defined as maturation-specific *Srf*-regulated genes (MGs), and genes down-regulated in both models were defined as constitutively *Srf*-regulated genes (CGs). A large majority of both MGs (657 of 931, 70.5%) and CGs (46 of 68, 67.6%) neighbored an SRF-bound region, suggesting that they were directly activated by *Srf*. We noted that mature sarcomere isoforms *Myh6*, *Myl3*, *Myl2*, and *Tnni3* were highly down-regulated in *Srf* KO, whereas their immature counterparts (*Myh7*, *Myl4*, *Myl7*, and *Tnni1*) were not (Fig. 3g). Interestingly, these four mature sarcomere isoform genes, but not their immature paralogs, were associated with strong SRF occupancy near their promoters (Fig. 4f). We investigated more broadly the relationship of SRF occupancy signal and TSS distance to differential gene expression. For MGs, both SRF occupancy signal and proximity to TSS were associated with the fraction of adjacent genes that were down-regulated with *Srf* KO; indeed, 78.2% (514/657) of all SRF-regulated MGs had SRF binding in the top six deciles and within 10 kb of the TSS (Fig. 4g). By contrast, this relationship was not observed for up-regulated genes (Supplementary Fig. 7d). Together, these data suggest that the widespread loss of SRF occupancy and reduction of SRF binding strength during CM maturation directly contribute to the maturation specificity of SRF-mediated transcriptional regulation.

### Hierarchical regulation of CM maturation by SRF

**Fig. 5:**
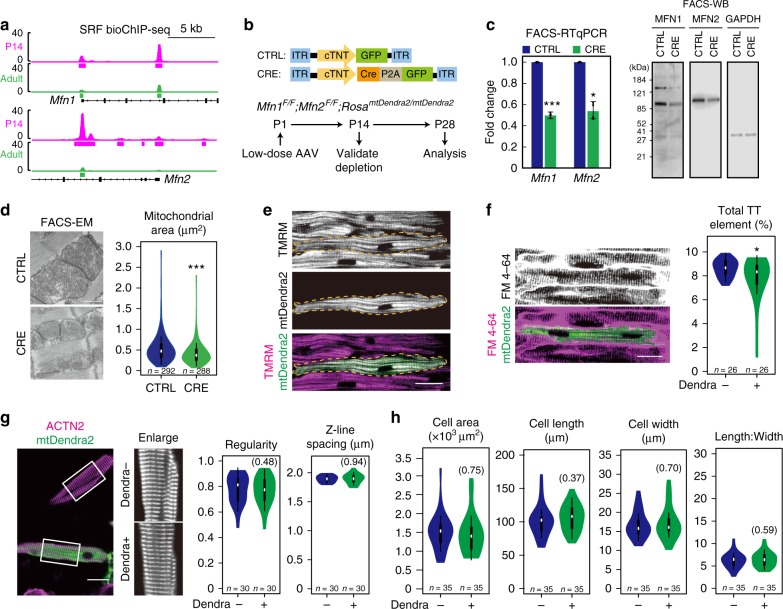
*Mfn1* and *Mfn2* play a minor role in CM maturation. **a** SRF bioChIP-seq signal showed proximal and distal chromatin binding near *Mfn1* and *Mfn2* genes specifically in actively maturing CMs. **b** Experimental design of mosaic, neonatal depletion of both MFN1 and MFN2. **c** Depletion of *Mfn1* and *Mfn2* mRNA (left) and protein (right) in FACS-sorted GFP/Dendra2+ CMs at P14. *Mfn1* and *Mfn2* mRNA expression was measured by RT-qPCR. MFN1 and MFN2 protein expression was measured by Western blotting. **d** FACS-EM analyses showed decreased mitochondria size upon *Mfn1/2* depletion. **e** Effect of MFN1/2 depletion on mitochondrial organization. In situ imaging of TMRM staining. **f** Effect of MFN1/2 depletion on T-tubule morphology. In situ T-tubule imaging was quantified by AutoTT. **g** Effect of MFN1/2 depletion on sarcomere organization. **h** Effect of MFN1/2 depletion on cell size and geometry. Violin plots are described in Fig. 2. Non-significant *P* values are shown within parentheses. Bar plots show mean ± SD and are overlaid by dot plots. Scale bar, 20 μm. Two-tailed Student’s *t* test: *P < 0.05, ***P < 0.001

To connect SRF-based transcriptional regulation with morphological and functional phenotypes that were observed in SRF-depleted cells (Fig. 2), we next studied the roles of direct SRF downstream genes in CM maturation. Mitochondrial fusion regulators *Mfn1/2* are essential for heart development at perinatal stages^32^. SRF bound to both proximal and distal regions near *Mfn1/2* in a maturation-specific manner (Fig. 5a). In the neonatal *Srf* KO model, *Mfn1* and *Mfn2* were down-regulated (Fig. 3f) and mitochondrial size decreased (Supplementary Fig. 5a), a typical phenotype of defective mitochondrial fusion. Therefore, we hypothesized that *Mfn1/2* were direct SRF targets that played a key role in CM maturation.

To probe the cell-autonomous contribution of *Mfn1/2* on CM maturation, we inactivated *Mfn1/2* in a small fraction (~15%) of CMs by injecting low-dose AAV-Cre-P2A-GFP (CRE) or AAV-GFP (CTRL) into P1 *Mfn1*^*F/F*^*;Mfn2*^*F/F*^*;Rosa*^*mtDendra2*^ mice (Fig. 5b), which harbored well-characterized floxed alleles of *Mfn1/2*^33^. FACS-sorted AAV-transduced Dendra/GFP+ CMs exhibited depletion of *Mfn1* and *Mfn2* RNA and protein in the CRE group compared to CTRL group (Fig. 5c). Mitochondria fusion defects in the mutant CMs were further validated by decreased mitochondria size through EM analysis (Fig. 5d). Surprisingly, MFN1/MFN2-depleted CMs exhibited very mild T-tubule disorganization and no detectable disruption of TMRM labeling, sarcomere organization, cell size, or shape (Fig. 5e–h). To further confirm this result, we overexpressed DRP1, a key activator of mitochondrial fission^34^, through AAV-based gene transfer in neonatal CMs (Supplementary Fig. 8a, b). This approach up-regulated DRP1 by more than 10-fold (Supplementary Fig. 8c) and decreased mitochondria size (Supplementary Fig. 8d). Despite these strong perturbations to mitochondrial dynamics and morphology, we observed very mild effects on mitochondria membrane potential, T-tubule formation, sarcomere organization, cell size, and shape (Supplementary Fig. 8e–h). These findings are consistent with our recent study of *Tfam*^35^, a critical mitochondrial transcription factor that was down-regulated upon SRF depletion (Fig. 2f), as well as a recent DRP overexpression study using transgenic mice^36^. The striking difference between our mosaic analyses and previous organ-wide ablation of *Mfn1/2* in CMs suggests that secondary effects of heart failure confounded studies of organ-wide *Mfn1/2* cardiac KO^32^. Together, our data indicate a minor role of mitochondrial dynamics in other aspects of CM maturation.

**Fig. 6:**
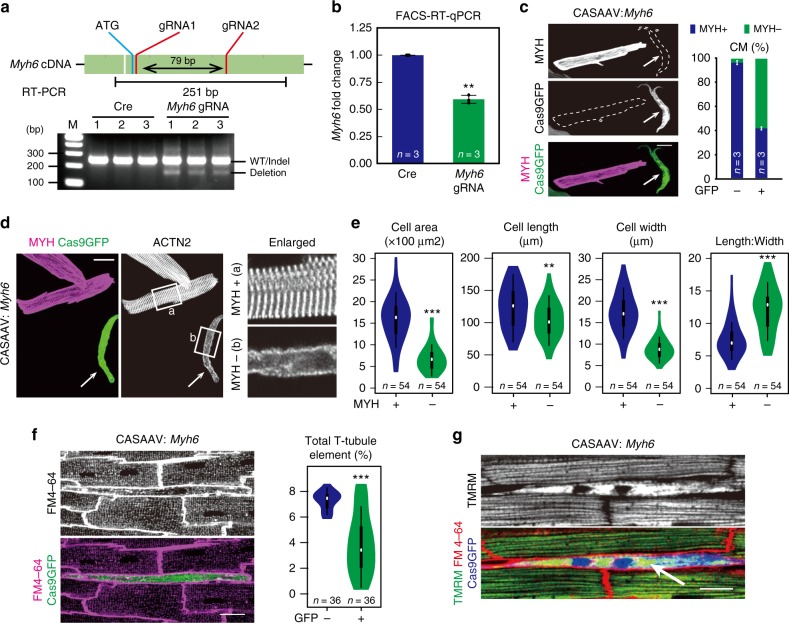
Sarcomere assembly is essential for other aspects of CM maturation. **a** CASAAV ablation of *Myh6*. Dual gRNAs targeting the first coding exon of *Myh6* gene induced Cas9-mediated deletion of the intervening genomic region when delivered to P1 CMs using the CASAAV system. Deletion validation and phenotypic analyses were performed at P14 and 1 month after injection, respectively. Deletion was monitored by RT-PCR. **b** Validation of *Myh6* depletion. After CASAAV-based neonatal *Myh6* mutagenesis, FACS-sorted GFP+ CMs at P14 were used to quantify *Myh6* by RT-qPCR. **c** Identification of individual CMs with successful MYH6 depletion by myosin heavy chain (MYH) immunofluorescence staining (arrow). Cell boundaries were delineated by dashed lines. The fraction of cells that were depleted of MYH was quantified to the right. **d** Sarcomere loss in CMs depleted of MYH6. After *Myh6* CASAAV, dissociated CMs were stained for ACTN2. Arrow points to a MYH-depleted CM. Boxed regions are enlarged to the right. **e** MYH6 depletion disrupted maturational hypertrophic growth of CMs. MYH6-depleted CMs were identified by immunostaining. **f** MYH6 depletion disrupted T-tubulation. T-tubule organization was measured by in situ imaging and AutoTT quantification. Representative image shows defective T-tubules within a Cas9GFP+ cell. **g** MYH6 depletion caused mitochondrial disorganization. Mitochondria were imaged in situ by TMRM staining. GFP− cells (no blue pseudocolor) had highly organized arrays of mitochondria, unlike the mitochondrial staining pattern in GFP+ cells (arrow). Scale bars, 20 μm in all images. Violin plots are described in Fig. 2. Bar plots show mean ± SD and are overlaid by dot plots. Numbers in bar indicate sample size. Two-tailed Student’s *t* test: **P < 0.01, ***P < 0.001

Another key facet of CM maturation downstream of *Srf* was sarcomere assembly. Therefore, we next probed the contribution of sarcomere assembly to overall CM maturation. We inactivated *Myh6*, a direct *Srf* target (Figs. 3g and 4f) that composed the majority of myosin heavy chains in mature myofibrils, by CASAAV. At P1, we delivered the CASAAV virus, containing two gRNAs targeting *Myh6* sites separated by 79 bp, to *Rosa*^*Cas9GFP*^ mice (Fig. 6a). This resulted in detectable deletion of the 79 bp fragment (Fig. 6a). We titrated the dose to achieve mosaic MYH6 ablation without impacting heart contraction. Depletion of *Myh6* mRNA and protein was further validated by FACS-RT-qPCR and immunostaining, respectively (Fig. 6b, c). MYH6 ablation caused complete disassembly of sarcomeres (Fig. 6d) as well as dramatic defects in maturational hypertrophy, T-tubulation, and mitochondrial organization (Fig. 6e–g). Together, these data show that sarcomeres are core organizers of other aspects of CM maturation. The diverse CM maturation events are orchestrated in a hierarchical manner that requires myofibrillar maturation.

### Balanced SRF activity is essential for CM maturation

**Fig. 7:**
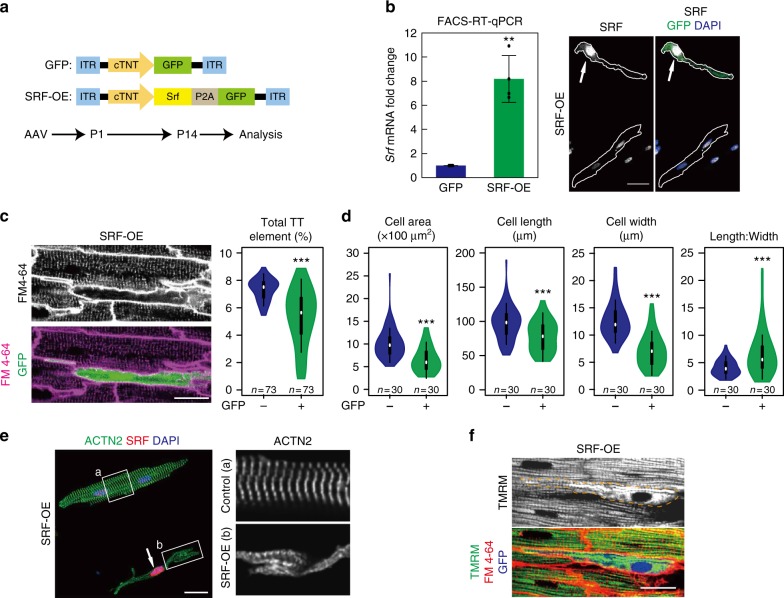
SRF overexpression perturbs CM maturation. **a** Schematic design of AAVs overexpressing SRF (SRF-OE) or GFP (control). AAVs were injected into P1 pups. Analysis of maturation was performed at P14. **b** RT-qPCR of FACS-sorted P14 GFP+ CMs validated *Srf* overexpression (left, *n* = 4 hearts). Immunofluorescence confirmed nuclear localization of overexpressed SRF proteins in GFP+ CMs (right). Cell boundaries are delineated by white lines. **c** SRF overexpression disrupted T-tubulation. T-tubule organization was measured by in situ imaging (left) and AutoTT quantification (right). SRF-overexpressed CMs were labeled by GFP. **d** SRF overexpression disrupted maturational hypertrophic growth of CMs. **e** Sarcomere disorganization in CMs overexpressing SRF. Dissociated CMs were stained for ACTN2. Boxed areas are enlarged (right). Arrow points to a validated SRF (red) overexpressing CM. **f** SRF overexpression caused mitochondrial disorganization. Mitochondria were imaged in situ by TMRM staining. GFP− cells (no blue pseudocolor) had highly organized arrays of mitochondria, unlike the mitochondrial staining pattern in GFP+ cells (cell boundary delineated). Scale bars, 20 μm in all images. Violin plots are described in Fig. 2. Bar plots show mean ± SD and are overlaid by dot plots. Two-tailed Student’s *t* test: **P < 0.01, ***P < 0.001

Given the profound impact of *Srf* on CM maturation, we wondered whether activating *Srf* was sufficient to promote CM maturation. To answer this, we overexpressed SRF in neonatal CMs through mosaic AAV-mediated gene delivery (Fig. 7a). GFP overexpression was used as CTRL. This approach up-regulated *Srf* by ~8-fold in transduced CMs, as measured by FACS-RT-qPCR. Nuclear accumulation of SRF was validated by immunofluorescence (Fig. 7b). Strikingly, SRF overexpression dramatically disrupted T-tubule formation, maturational hypertrophy, sarcomere organization, and mitochondria distribution (Fig. 7c–f).

**Fig. 8:**
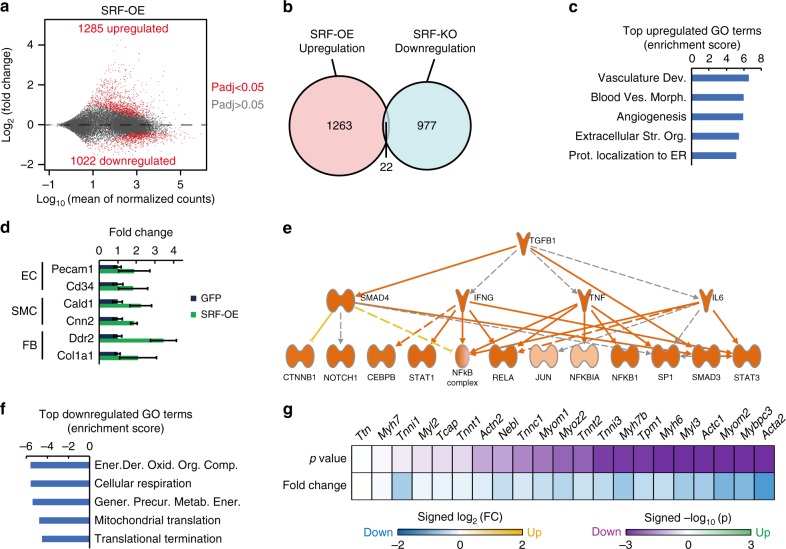
SRF overexpression perturbs the transcriptional program in maturing CMs. **a** SRF overexpression and control P14 CMs were FACS sorted and analyzed by RNA-seq. MA plots showed dramatic transcriptional dysregulation. Significantly dysregulated genes (*P*adj < 0.05) were dots in red. **b** Up-regulated genes upon SRF overexpression showed little overlap with genes down-regulated upon *Srf* KO at the same stage, as viewed by Venn diagram. **c** Gene ontology terms enriched among genes up-regulated upon SRF overexpression. The GSEA normalized enrichment scores for the top 5 terms were plotted. **d** Selected markers of endothelial cells (EC), smooth muscle cells (SMC), and fibroblasts (FB) that were up-regulated by SRF overexpression. **e** IPA upstream regulator analysis showed up-regulation of inflammatory response pathways upon SRF overexpression. The color of the lines signifies the expected direction of effect between two nodes. Blue represents predicted inhibition and orange represents predicted activation. Yellow signifies inconsistency between the gene expression in the data set and the annotated relationship. Gray indicates no prediction. Solid and dashed lines indicate direct and indirect interactions, respectively. **f** Gene ontology terms enriched among genes down-regulated upon SRF overexpression. **g** Statistical analyses of the differential expression of major sarcomere genes. Fold changes and *P* values were plotted in blue-yellow and magenta-green color scales, respectively. Differential expression analysis *P* values were calculated by DESeq2^59^

We next analyzed transcriptomic changes by administering a mosaic dose of either AAV-SRF-P2A-GFP or AAV-GFP, purifying transduced GFP+ CMs by FACS and then performing RNA-seq. SRF overexpression up-regulated 1285 genes (Fig. 8a). Interestingly, very few of these genes (1.7%) overlapped with genes down-regulated by SRF KO (Fig. 8b). By GSEA analysis, vasculature development and angiogenesis were the major up-regulated GO terms in SRF overexpressing CMs (Fig. 8c). Consistent with this observation, the up-regulated genes included markers of endothelial cells, smooth muscle cells, and fibroblasts (Fig. 8d). Through an independent IPA analysis, we also identified the activation of inflammatory signaling pathways involving transforming growth factor-β1, interferon-γ, tumor necrosis factor, and interleukin-6. (Fig. 8e). Thus, SRF overexpression causes ectopic gene activation that should not be present in maturing CMs. Furthermore, SRF overexpression caused dramatic down-regulation of metabolism and myofibril genes (Fig. 8f, g), which explained the defects in morphological maturation (Fig. 7). Together, these data indicate that SRF activity must be carefully balanced for proper CM maturation.

## Discussion

**Fig. 9:**
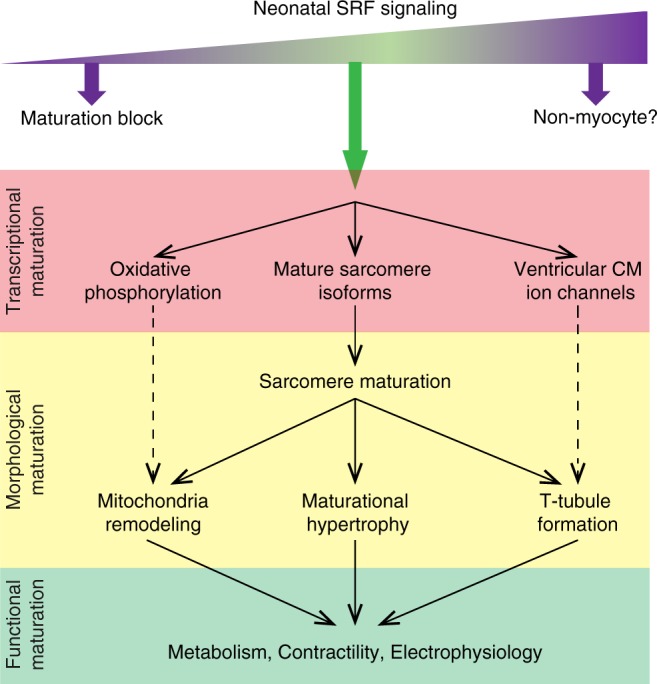
Schematic summary of SRF regulation of CM maturation. A balanced level of SRF activity is required in maturing CMs for the transcription of major genes that are required for CM maturation. These genes include those that regulate mitochondria and metabolism, sarcomere assembly, and ion channels. Among these genes, sarcomere genes are particularly critical for organizing not only myofibrils but also other aspects of morphological maturation, such as mitochondria remodeling, maturational hypertrophy, and T-tubule formation. These coordinated transcriptional and morphological maturation events collectively establish the robust functions of adult CMs, which is maintained throughout the animal’s lifespan

CM maturation is one of the least understood processes in heart development. In this study, we performed a CRISPR/Cas9-based screen in mice and identified SRF as a transcriptional regulator that orchestrated almost every aspect of CM maturation. Using SRF as a model molecule, we uncovered several critical principles that govern CM maturation (Fig. 9): First, SRF regulated maturation only in actively maturing, neonatal CMs through stage-specific chromatin occupancy and transcriptional control. This implies the presence of a unique, maturation-specific transcriptional regulation network that was not recognized previously. Second, we showed that SRF signaling needs to be tightly balanced for proper maturation. Both hypo-activation and hyper-activation of SRF resulted in severe transcriptional dysregulation that impacted sarcomere and mitochondria maturation. Third, the diverse maturation processes downstream of SRF appear to be orchestrated in a hierarchical manner. That sarcomere inactivation was sufficient to impair multiple facets of CM maturation suggests that myofibrillar maturation is a dominant and essential process, and that sarcomeres are core organizers of other aspects of CM maturation. However, our data do not exclude additional direct roles of SRF in other aspects of maturation, such as mitochondrial maturation. Overall, this report provides direct demonstration of an essential and central role of sarcomeres in organizing the diverse programs of CM maturation.

The new information acquired in this study provides potential guidance to mature stem cell-derived CMs. For example, biomechanical environments are known to influence the maturation of CMs in vitro. Specifically, an intermediate stiffness of cell culture matrix, mimicking the native mechanical environment, was reported to be essential for CM maturation^37,38^. SRF is a well-established effector of mechanotransduction signaling in response to matrix stiffness^39,40^; thus, an intermediate matrix stiffness might promote CM maturation by establishing an intermediate level of SRF activity. In addition, sarcomere disarray is a common phenotype that is observed in CMs cultured in a monolayer on an unpatterened substrate. Culture on micropatterned substrates^41–43^ or within three-dimensional substrates with directional tension^44–47^ improve sarcomere organization. Our results demonstrate that organized sarcomere assembly is a key organizer on top of the hierarchy of other CM maturation processes. These results indicate that sarcomere organization achieved by these engineered environments is essential to enhance all other aspects of CM maturation, and that optimization of SRF signaling and sarcomere organization are likely to be key mechanisms by which bioengineering approaches improve maturation of stem cell-derived CMs. These insights promise to allow us to use rationale approaches to further optimize the maturation of stem cell-derived CMs.

SRF is a well-established cardiac TF that has been studied for more than a decade in the heart. However, prior studies used traditional conditional KO strategies that cause acute lethality and cardiomyopathy^12–14,16,48^. As a result, the critical role of SRF in CM maturation was overlooked. Here we establish AAV-based genetic mosaic analyses as a key strategy to minimize confounding secondary effects of heart dysfunction. This generated new information that significantly updated our prior understanding of the function of SRF in CMs. For example, although we observed sarcomere defects that agreed with SRF’s previously established function, neonatal and mosaic SRF KO exhibited grossly normal striated myofibril patterns—a much milder phenotype than previously reported in SRF-depleted developing hearts^12,48^. Prior studies found that SRF was required to activate transcription of both mature and immature sarcomere components^21^. However, here we demonstrated a selective role of SRF in activating only mature, but not immature, sarcomere isoforms. In addition, we found dramatic down-regulation of GO terms related to mitochondria and metabolism, but not heart development or muscle cell differentiation, in neonatal mosaic *Srf* KO. This profound role of SRF in mitochondria and metabolism was not observed in previous studies, likely due to both acute lethality that precluded study of Srf at the neonatal stage and to obfuscating effects of heart failure. Likewise, the mosaic strategy allowed us to circumvent lethality caused by *Myh6* disruption and thereby hone in on the essential function of sarcomeres to promote CM maturation.

Our genetic mosaic analysis also challenges the established paradigm of mitochondrial dynamics in heart development. We observed minimal morphological phenotypes upon mosaic MFN1/2 depletion or DRP1 overexpression in neonatal CMs. This finding contrasts sharply with the dramatic heart phenotypes of conventional organ-wide *Mfn1/2* double KOs^32,49^. This is likely due to secondary effects of heart dysfunction that amplified the severity of the phenotypes in previous *Mfn1/2* double KOs, although we cannot rule out the possibility that *Mfn1/2* are required for CM maturation at an earlier embryonic stage, or that the kinetics of *Mfn1/2* inactivation in our system missed a critical time window necessary to observe the more dramatic effects that were previously reported. This study, together with previous studies of *Tfam*^35^ and *Drp1*^36^, indicates a relatively minor role of normal mitochondria function in promoting CM maturation.

## Methods

### Mouse strains

All animal strains and procedures were approved by the Institutional Animal Care and Use Committee of Boston Children’s Hospital.

Rosa^Cas9GFP/Cas9GFP^(Jackson Lab Stock No.: 026175)^50^, Rosa^Tomato/Tomato^(Jackson Lab Stock No.: 007914)^51^, Rosa^mTmG/mTmG^(Jackson Lab Stock No.: 007576)^52^, and Rosa^BirA/BirA^ (Jackson Lab Stock No.: 010920)^26^ were imported from the Jackson Laboratory. Srf^F/F^^12^ (Jackson Lab Stock No.: 006658), Mfn1^F/F^;Mfn2^F/F^;Rosa^mtDendra2^ ^33,53^ (Jackson Lab Stock No.: 026401, No.: 026525, and No.: 018385), and Tbx5^F/F^ ^11^ mice were kind gifts from the labs of Joe Miano, David Chan, and Ivan Moskowitz. All mice were on a mixed genetic background.

Srf^fbio^ mice were generated by homologous recombination in ES cells (Extended Data Fig. 6a). A targeting vector containing homology arms, the FLAG-BIO epitope tag fused to the 3′ end of SRF, and an Frt-Neo-Frt selection cassette was used to generate targeted ES cells. Blastocyst injection yielded chimeric mice. Germline transmission through Actb-Flpe removed the Frt-Neo-Frt cassette. Actb-Flpe was subsequently removed by breeding. The mice are available at MMRRC, Stock No.: 37511.

### Plasmids

AAV-cTNT-Cre, AAV-U6gRNA-U6gRNA-cTNT-Cre, and AAV-cTNT-GFP plasmids^6,7,15^ are available at Addgene.

To generate AAV-cTNT-GFP-version2, a 63 bp multiple cloning site was synthesized as single-stranded oligos, annealed, and inserted into AAV-cTNT-GFP through *Nhe*I and *Nco*I sites. Next, 3XHA-P2A sequence was synthesized (IDT) and inserted into AAV-cTNT-GFP-version2 through *Nhe*I and *Nco*I sites to generate AAV-cTNT-3XHA-P2A-GFP. Cre coding sequence was PCR-amplified from AAV-cTNT-Cre and inserted into AAV-cTNT-3XHA-P2A-GFP through *Nhe*I and *Sac*I sites to generate AAV-cTNT-Cre-P2A-GFP. To generate AAV-cTNT-SRF-P2A-GFP, Srf cDNA was purchased from GE Healthcare Dharmacon Inc. (# MMM1013-202798340), amplified by PCR, and inserted into AAV-cTNT-GFP-version2 plasmid at *Nhe*I and *Spe*I sites. The new plasmids will be available at Addgene.

For CASAAV-mediated gene depletion, we designed 1–2 gRNAs per target gene using the GPP Web Portal (Broad Institute). The gRNA sequences were synthesized as single-stranded oligos, annealed, and inserted into AAV-U6gRNA-U6gRNA-cTNT-Cre plasmids^6,7^ at *Sap*I and/or *Aar*I sites. gRNA sequences that were used in this study are summarized in Supplementary Table 1.

### AAV production and injection

One hundred and forty micrograms of AAV-ITR, 140 µg AAV9-Rep/Cap, and 320 µg pHelper (pAd-deltaF6, Penn Vector Core) plasmids were produced by Maxiprep (Invitrogen, K210017) and transfected into 10 15-cm plates of HEK293T cells using PEI transfection reagent (Polysciences, 23966-2). Sixty hours after transfection, cells were scraped off of plates, resuspended in lysis buffer (20 mM Tris, pH 8, 150 mM NaCl, 1 mM MgCl_2_, 50 µg/ml benzonase) and lysed by three freeze-thaw cycles. AAV in cell culture medium was precipitated by PEG 8000 (VWR, 97061-100), resuspended in lysis buffer, and pooled with cell lysates. AAV was purified in a density gradient (Cosmo Bio USA, AXS-1114542) by ultracentrifugation (Beckman, XL-90) with a VTi-50 rotor and concentrated in phosphate-buffered saline (PBS) with 0.001% pluronic F68 (Invitrogen, 24040032) using a 100 kDa filter tube (Fisher Scientific, UFC910024). AAV titer was quantified by qPCR (primer sequences in Supplementary Table 2) using a fragment of the TNT promoter DNA to make a standard curve.

AAV was injected into P1 pups subcutaneously. The P1 pups were anesthetized in an isoflurane chamber before injection. Intraperitoneal injection was performed to inject AAV into adult animals. AAV dosage was normalized based on body weight at both neonatal and adult stages. In total, 5 × 10^8^ viral genome per gram body weight (vg g^−1^) was used in all mosaic analyses in this study. High and intermediate doses corresponded to 1 × 10^10^ vg g^−1^ and 5 × 10^9^ vg g^−1^, respectively.

### Echocardiography

Echocardiography was performed on a VisualSonics Vevo 2100 machine with the Vevostrain software. Animals were awake during this procedure and held in a standard handgrip. The echocardiographer was blinded to genotype and treatment.

### Histology

After animals were euthanized by CO_2_. Hearts were harvested immediately and fixed by 4% paraformaldehyde overnight at 4 °C. Fixed hearts were cryoprotected by soaking in 15% sucrose followed by 30% sucrose at 4 °C. Hearts were embedded in tissue freezing medium (General Data, TFM-5). Ten micrometers of cryo-sections were cut using a cryostat (Thermo Scientific, Microm HM 550).

For Fast Green and Sirus Red staining, the frozen sections were washed with PBS for 5 min, fixed with pre-warmed Bouin’s solution (Sigma, HT10132) at 55 °C for 1 h, and washed in running water. The sections were next stained with 0.1% Fast Green (Millipore, 1040220025) for 10 min, washed with 1% acetic acid for 2 min, and rinsed with running water for 1 min. The sections were next stained with 0.1% Sirus Red (Sigma, 365548) for 30 min and washed with running water for 1 min. The slides were treated with 95% ethanol once for 5 min, twice with 100% ethanol for 5 min, and twice in xylene for 5 min before being mounted with Permount (Fisher Scientific, SP15-500). Bright-field images of stained tissue sections were taken under a dissection microscope (Zeiss, SteREO Discovery V8).

### In situ confocal imaging

In situ T-tubule imaging was performed as previously described^6,10^. In brief, hearts were dissected from euthanized animals and cannulated on a Langendorff apparatus. FM 4–64 (2 µg/ml) (Invitrogen, 13320) was diluted in perfusion buffer (10 mM HEPES (pH 7.4), 120.4 mM NaCl, 14.7 mM KCl, 0.6 mM KH_2_PO_4_, 0.6 mM Na_2_HPO_4_, 1.2 mM MgSO_4_, 4.6 mM NaHCO_3_, 30 mM taurine, 10 mM 2,3-butanedione monoxime, 5.5 mM glucose) and loaded into the heart by retrograde perfusion at room temperature for 10 min. The heart was next removed from the perfusion system, positioned on a glass-bottom dish, and immediately imaged on an inverted confocal microscope (Olympus FV1000).

In situ mitochondria imaging was performed by loading both 2 nM TMRM (mitochondrial marker) and 2 µg/ml FM 4–64 (cell membrane marker) into the heart by retrograde perfusion at room temperature for 10 min. The heart was imaged on an inverted confocal microscope (Olympus FV1000).

### CM isolation

CMs were isolated by retrograde collagenase perfusion using an established protocol^54^. In brief, heparin-injected mice were anesthetized in an isoflurane chamber. Hearts were isolated and cannulated onto a Langendorff perfusion apparatus. Perfusion buffer (at 37 °C) was first pumped into the heart to flush out blood and equilibrate the heart. Collagenase II (Worthington, LS004177) was next perfused into the heart for 10 min at 37 °C to dissociate CMs. Heart apex was cut from the digested heart, gently dissociated into single CMs in 10% fetal bovine serum (FBS)/perfusion buffer, and filtered through a 100 µm cell strainer to remove undigested tissues.

### Immunofluorescence

To prepare cells for immunofluorescence, the isolated CMs were concentrated by 20 × *g* centrifugation for 5 min and resuspended in the cell culture medium (Dulbecco's modified Eagle's medium (Gibco), 10% FBS, pen/strep (Gibco), 10 µM blebbistatin). CMs were cultured on laminin-coated coverslips for ~40 min at 37 °C with 5% CO_2_ to allow cells to attach to the coverslips.

Next, immunofluorescence was performed following published protocols^6,7,55,56^. In brief, CMs were fixed on coverslips by 4% paraformaldehyde for 10–20 min, permeabilized by 0.1% Triton-100/PBS for 10 min, and blocked in 4% bovine serum albumin/PBS (blocking buffer) at 4 °C overnight. Then, the cells were incubated with primary antibodies diluted in blocking buffer overnight at 4 °C. After washes with blocking buffer, the cells were incubated with secondary antibodies and dyes at room temperature for 2 h. The cells were next washed with PBS and mounted with ProLong Diamond antifade mountant (Invitrogen, 36961) before imaging. All antibodies and dyes are listed in Supplementary Tables 3 and 4.

TUNEL staining was performed using In Situ Cell Death Detection Kit (Roche Diagnostics, #11684795910) following the manufacturer’s instruction.

### Fluorescence imaging and analysis

Confocal fluorescence images were taken using Olympus FV1000 inverted laser scanning confocal microscope equipped with a ×60/1.3 silicone-oil objective. Fluorescence intensity was measured using ImageJ. AutoTT^9^ was used to quantify T-tubule and sarcomere organization. Total TT elements refer to the sum of longitudinal and transverse T-tubule elements. Cell size and shape was manually measured on maximally projected images.

### Contractility assay and calcium imaging

Before contractility and calcium analyses, calcium was re-introduced into isolated CMs by treating cells with a series of 10 ml 2,3-butanedione monoxim-free perfusion buffers containing 100 µM, 400 µM, 900 µM, and 1.2 mM CaCl_2_. At each step, CMs were allowed to settle by gravity for 10 min at room temperature before being transferred to the next buffer with higher calcium concentration.

For contractility assay, CMs were first settled in laminin-coated 6-well dishes at 30 °C for 10 min. FP− and FP+ cells were identified and imaged through epifluorescence microscope. Next, CMs were electrically stimulated at 1 Hz and cell contraction was recorded in the bright-field channel of a Keyence BZ-X700 microscope at 33 fps using a ×40 objective. SarcOptiM were used to quantify sarcomere shortening during contraction^18^.

For calcium imaging, CMs were loaded with 5 µM Fluo-4 (when FP reporter is Tomato) or Rhod-2 (when FP reporter is Cas9GFP) for 20 min. The cells were next washed with normal Tyrode solution (140 mM NaCl, 4 mM KCl, 1 mM MgCl_2_, 1.8 mM CaCl_2_, 10 mM glucose, 5 mM HEPES, pH = 7.4, adjusted with NaOH) for 20 min. The cells were next settled in a laminin-coated glass-bottom flow chamber at 30 °C for 10 min and electrically stimulated at 1 Hz to produce steady-state conditions. Calcium signals were next acquired through confocal line scanning using a ×60 objective. Line scan was positioned along the long axis of the cell in the cytosol, avoiding the nuclear area. Calcium signal was quantified manually using ImageJ.

### EM analysis after FACS

EM analysis after FACS (FACS-EM) was performed as follows. Isolated CMs in suspension were fixed with 4% paraformaldehyde for 30 min at room temperature. The fixed cells were next filtered by passing through a 100 µm cell strainer, pelleted by centrifugation at 20 × *g* for 5 min at room temperature, and resuspended in ~1 ml perfusion buffer. FACS was performed using a BD Aria II SORP cell sorter with a 100 µm nozzle. After FACS, the cells were fixed again in a mixture of 2% formaldehyde and 2.5% glutaraldehyde in 0.1 M sodium cacodylate buffer, pH 7.4, overnight at 4 °C. The cell pellets were next processed through a routine transmission EM (TEM) protocol at Harvard Medical School EM core. Images were taken using a JEOL 1200EX-80 kV EM. Because of the cell size and stiffness, fixed adult CMs easily clogged the FACS machine. Currently, FACS-EM only works for CMs from P30 and younger mice.

### Reverse transcription-quantitative PCR analysis

For regular RT-qPCR analysis, total RNA was purified using PureLink RNA Mini Kit (Ambion, 12183025). Genomic DNA removal and reverse transcription was performed using QuantiTech Reverse Transcription Kit (Qiagen, 205311). Real-time PCR was performed using an ABI 7500 thermocycler with Power SYBR Green PCR Kit (Thermo Fisher, 4368702). QPCR primers are listed in Supplementary Table 2.

For FACS-RT-qPCR, isolated CMs were filtered with a 100 µm cell strainer, pelleted by centrifugation at 20 × *g* for 5 min and resuspended in ~1 ml cold perfusion buffer. FACS were performed using a BD Aria II SORP cell sorter with a 100 µm nozzle and a sample collection cooling device. Immediately after FACS, cells were centrifuged at 13,000 rpm at 4 °C to remove supernatant. Total RNA was purified using PureLink RNA Micro Kit (Thermo Fisher, 12183016) and genome DNA removed by on-column DNase I digestion. RT was performed using SuperScript III Kit (Thermo Fisher), or SMART-Seq v4 Ultra Low Input RNA Kit (Clontech) if RNA yield was too low to be detected by regular RT-qPCR. Real-time PCR was performed using an ABI 7500 thermocycler using Taqman probes listed in Supplementary Table 5.

### Western blot analysis after FACS

FACS-sorted CMs were lysed in 2× sodium dodecyl sulfate sample buffer at 1000 cell/µl to normalize protein content. After boiling for 5 min, 5 µl cell lysate of each sample was separated on a 4–12% gradient gel (Invitrogen, Bolt gels, NW04122BOX), transferred to a polyvinylidene difluoride membrane, and blocked by 4% milk/TBST (Tris-buffered saline, 0.1% Tween-20). Primary antibodies were incubated with the membrane overnight at 4 °C, followed by four 15 min TBST washes. Horse radish peroxidase (HRP)-conjugated secondary antibodies were probed for 1–2 h at room temperature, followed by four 15 min TBST washes. After adding Immobilon Western Chemiluminescent HRP Substrate (Millipore, WBKLS0500), chemiluminescence were detected by a Li-Cor C-DiGit blot scanner. Antibodies used in this study are listed in Supplementary Table 3. All uncropped western blots can be found in Supplementary Fig. 9.

### RNA-seq and data analysis

FACS-sorted CMs were centrifuged at 10,000 × *g* for 1 min and supernatant fluids were removed. Total RNA was extracted using PureLink RNA Micro Kit (Thermo Fisher, 12183016) with genome DNA removed through on-column DNase I digestion. Ten nanograms of total RNA was reverse transcribed and full-length cDNA was specifically amplified by eight PCR cycles using SMART-Seq v4 Ultra Low Input RNA Kit (Clontech)^19^. RNA-seq libraries were constructed using Illumina’s Nextera XT Kit and single-ended reads were sequenced using NextSeq 500 sequencer at Harvard Medical School biopolymers facility.

RNA-seq reads were aligned to mm10 by STAR^57^ and reads counts were calculated by FeatureCounts^58^. DESeq2 was next used to perform statistical analysis of differential gene expression^59^. An adjusted *P* value of 0.05 was used as cutoff to identify differentially regulated genes. GSEA analysis with ranked gene lists was used to perform GO term analysis^60^. IPA (Qiagen Inc.) was used for upstream regulator network analysis^61^.

### BioChIP-Seq and data analysis

For each biological replicate, four heart ventricles were collected from two male and two female P14 Srf^fbio/+^;Rosa26^birA/+^ mice and minced in 1% formaldehyde-containing PBS at room temperature by a motor-driven homogenizer (IKA T10 basic). The tissue suspension was shaken for 15 min at room temperature for crosslinking. Glycine was added to final concentration of 125 mM to quench formaldehyde. Chromatin isolation was performed as previously described^62^. A microtip sonicator (QSONICA Q700) was used at 60% amplitude and a cycle of 5 s on and 20 s off for 96 cycles in total. Sheared chromatin was precleared by incubation with 100 µl Dynabeads Protein A (Life Technologies, 10002D) for 1 h at 4 °C. The precleaned chromatin was then incubated with 100 µl Dynabeads M-280 Streptavidin (Life Technologies, 11206D) for 1 h at 4 °C. The streptavidin beads were washed and bound DNA eluted^62^. BioChIP DNA was purified with MinElute PCR Purification Kit (Qiagen, 28006). ChIP-seq libraries were constructed using a ChIP-seq Library Preparation Kit (KAPA Biosystems, KK8500). Fifty nanograms of sonicated chromatin without pull-down was used as input.

Single-end sequencing (75 nt) was performed on a NextSeq 500 sequencer. Reads were aligned to mm10 using Bowtie 2^63^ using default parameters. Peaks were called with MACS2^64^ against input chromatin background. Murine blacklist regions were masked out of peak lists. Homer (http://homer.ucsd.edu/homer/) was used to annotate peaks to the nearest gene and to perform motif analysis^65^. DeepTools was used to generate aggregation and heatmap plots^66^. bioChIP-seq signal was visualized in the Integrated Genome Viewer^67^.

## Electronic supplementary material

Supplementary Information

Peer review file_new

## Data Availability

The authors declare that all data supporting the findings of this study are available within the article and its Supplementary information files or from the corresponding author upon reasonable request. RNA-seq and ChIP-seq data have been deposited in the Gene Expression Omnibus (GEO) database under the accession codes: GSE109425 (for the Srf KO RNA-seq), GSE109504 (ChIP-seq), and GSE116030 (for the Srf OE RNA-seq). The data are also available on the Cardiovascular Development Consortium server (https://b2b.hci.utah.edu/gnomex) (sign in as guest).
