# Supplementary material for: Hierarchical and stage-specific regulation of murine cardiomyocyte maturation by serum response factor
Source: Nat Commun. 2018 Sep 21;9:3837. doi: 10.1038/s41467-018-06347-2 (PMC6155060; doi:10.1038/s41467-018-06347-2)
Supplement: Supplementary file 1 — Supplementary Information [file 41467_2018_6347_MOESM1_ESM.pdf]

Hierarchical and stage-specific regulation of murine cardiomyocyte maturation by serum response factor

Guo et al.

Supplementary Information

9 Supplementary Figures

5 Supplementary Tables

Supplementary References

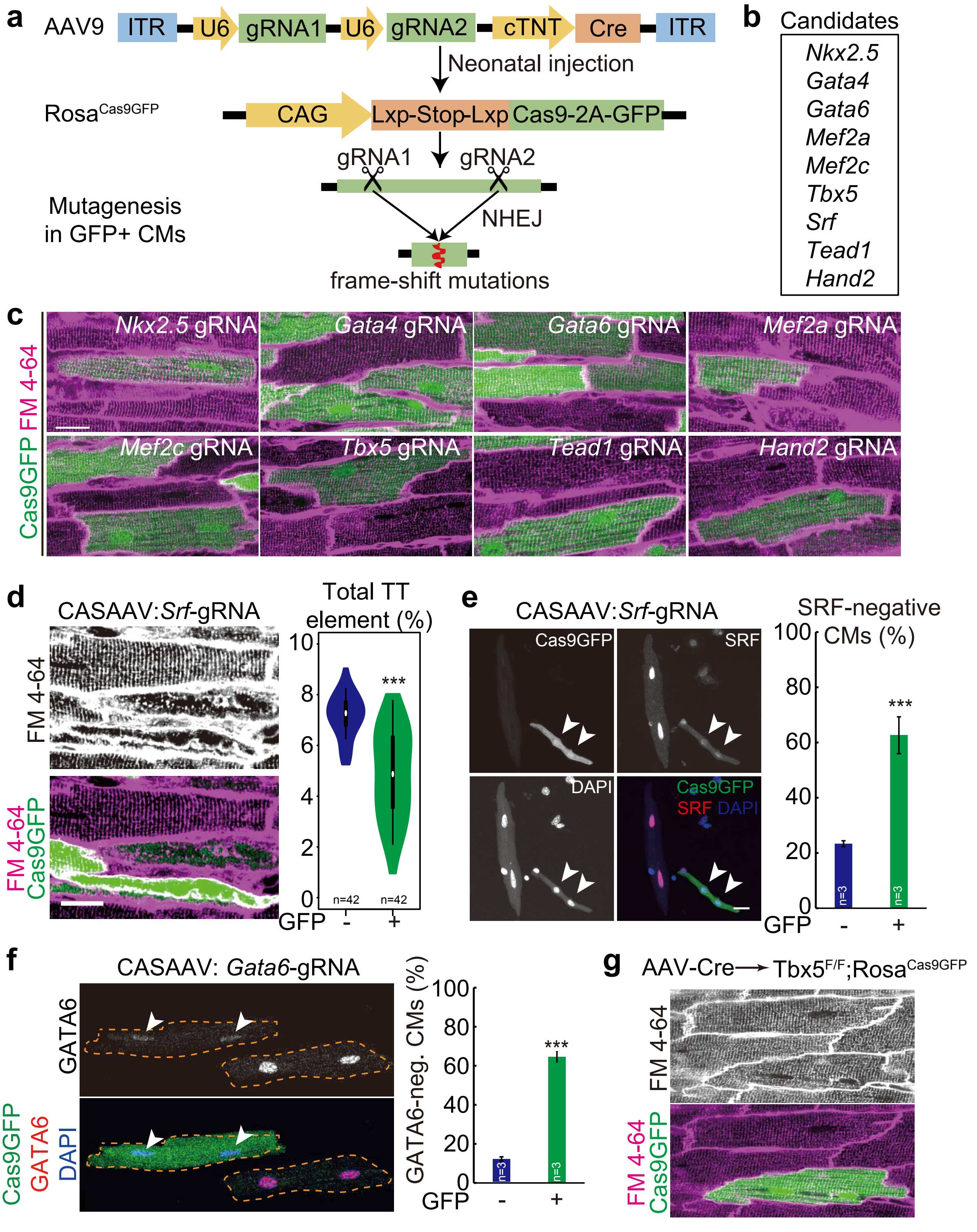

Supplementary Figure 1. CASAAV-based genetic screen identified an essential role of *Srf* in T-tubule maturation. a, Schematic representation of CASAAV based gene inactivation. AAVs were injected into P1 pups, phenotypic analyses performed one month later. **b,** Candidate cardiac TFs screened by CASAAV for effects on T-tubule maturation. **c,** Representative images of unaltered T-tubule organization upon treatment of newborn mice with CASAAV vectors targeting 8 candidates. **d,** CASAAV inactivation of Srf caused disruption of T-tubules (TT). **e,** Immunofluorescence validation of SRF depletion in Cas9GFP+ CMs by Srf-directed CASAAV vector. Arrows, GFP+ CM lacking SRF immunoreactivity in nuclei. **f,** Immunofluorescence validation of GATA6 depletion by CASAAV. **g**, Validation of a dispensable role of *Tbx5* in T-tubule formation using a established *Tbx5*$^{F/F}$ allele. CMs from 3 hearts were analyzed. Quantification was plotted as bar plots (mean ± SD) or violin plots (See figure 2). Two-tailed student's t-test: ***P<0.001.

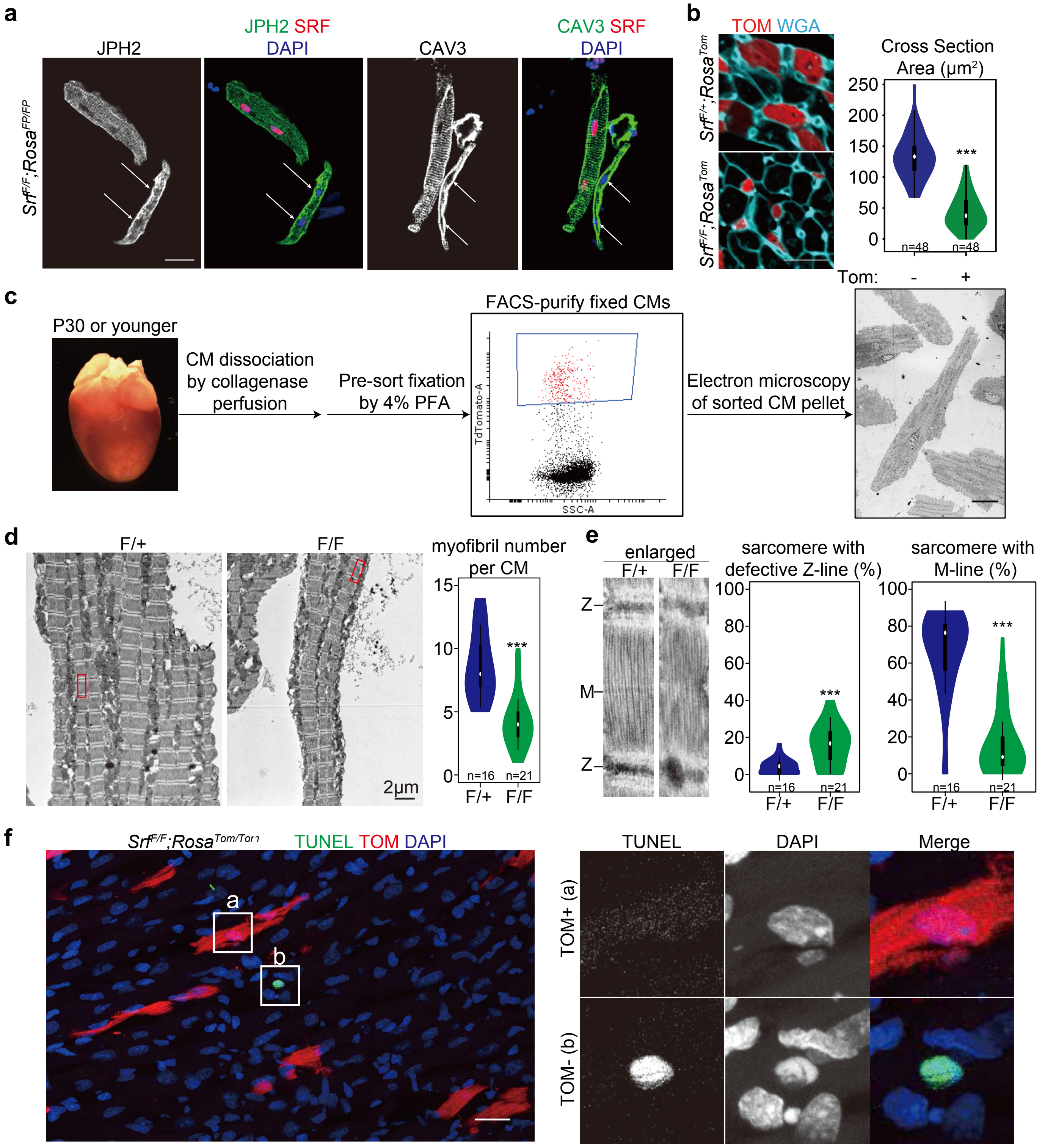

Supplementary Figure 2. *Srf* is essential for morphological CM maturation. a, Validation of T-tubule phenotypes in isolated *Srf* KO CMs by immunofluorescence of JPH2 and CAV3. Arrows indicated *Srf* KO CMs in which nuclei are depleted of SRF staining. **b**, Validation of cell size pheotypes in *Srf* KO CMs by measuring CM area on heart cross sections. **c-e,** Effect of SRF depletion on sarcomere organization, as determined by FACS-EM. **c**, Method to analyze FACS-sorted CMs by EM. **d-e,** Representative EM images of control and SRF-depleted CMs. Boxed regions in **d** are enlarged in **e**. The fractions of sarcomeres with or without M-lines were quantified per cell images and compared between control and mutant gourps in **e**. **f**, TUNEL staining showed no apoptosis in FP+ CMs on one-month-old heart tissue sections. Boxed regions are enlarged to the right. The TUNEL+ nucleus in a FP- cell in (b) shows that TUNEL staining works. Two-tailed student's t-test: **P<0.01, ***P<0.001. Scale bars are 20 μm unless otherwise labeled.

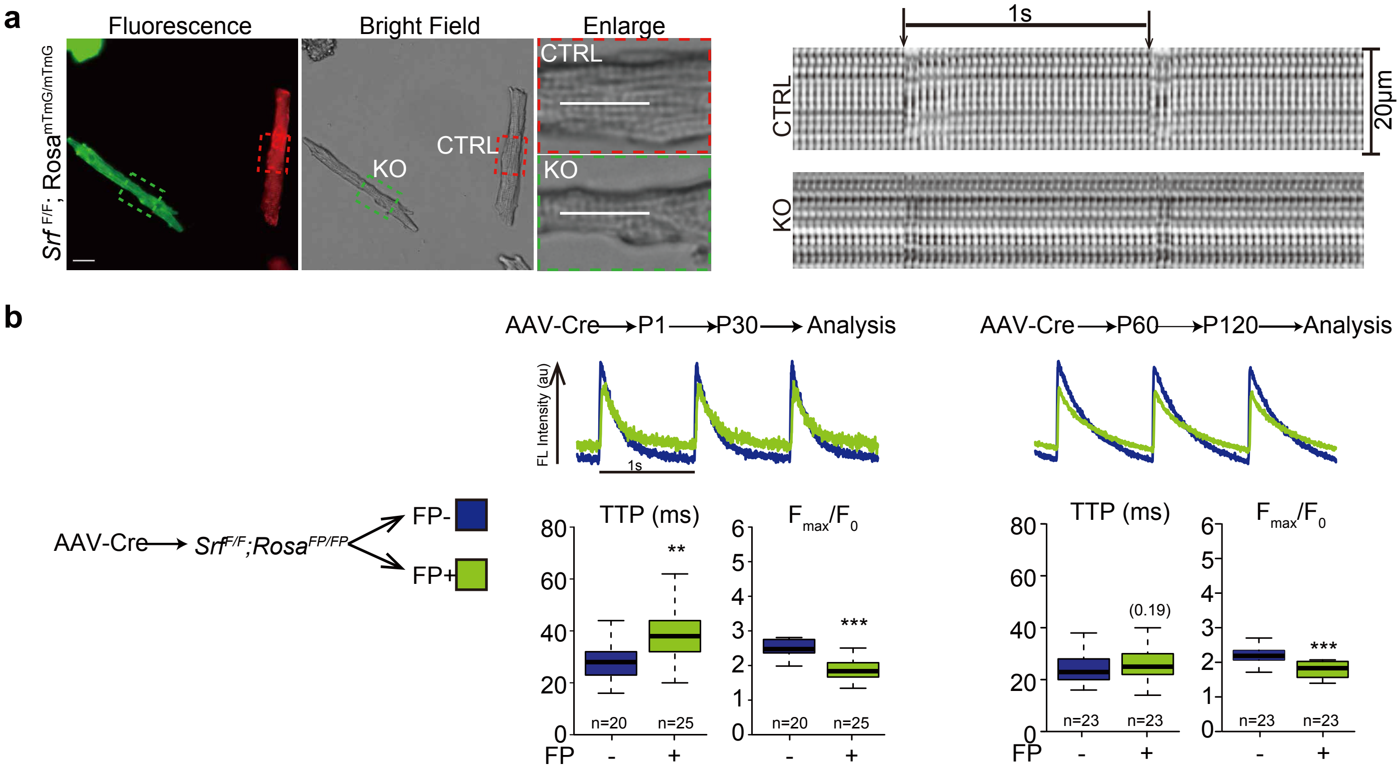

Supplementary Figure 3. *Srf* is essential for functional maturation of CMs. a, CM contraction assay. Representative epi-fluorescence and brightfield images illustrate classification of cells by FP expression. Boxed regions are enlarged in inset, where white lines indicate regions presented as kymographs to the right. **b,** Effect of neonatal or adult *Srf* KO on calcium transients using Calcium Dye Fluo-4 or Rhod-2. Quantification of time to peak calcium signal (TTP) and peak calcium intensity (F_{max}/F_0) are shown in box plots. In box plots, center lines and boxes indicate median and 25th and 75th percentiles. Whiskers extend 1.5 times the interquartile range from the 25th and 75th percentiles. Two-tailed student's t-test: **P<0.01, ***P<0.001.

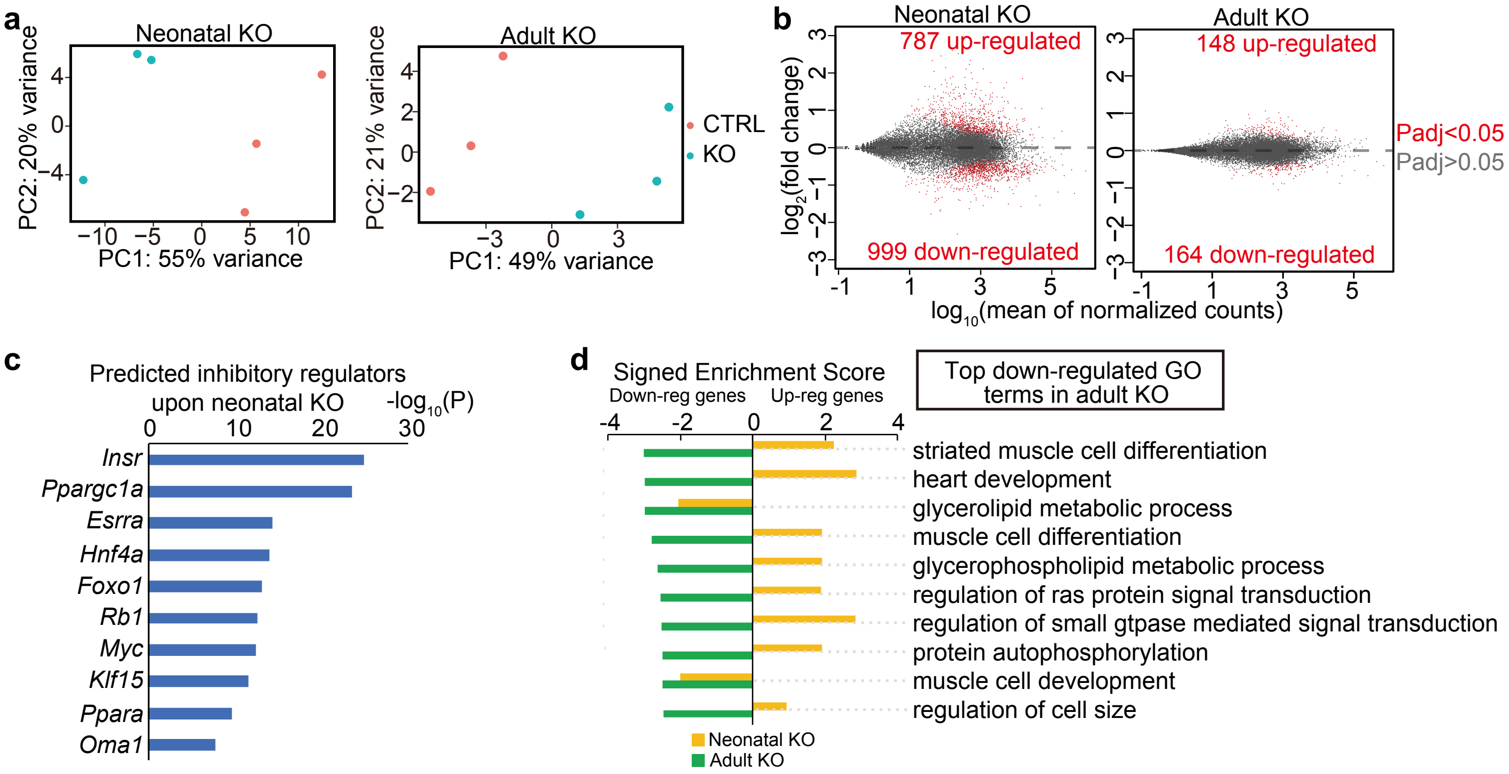

Supplementary Figure 4. Maturation-specific transcriptional regulation by *Srf*. a, Individual PCA plots of RNA-seq data in *Srf* neonatal or adult KO models. **b,** MA plots of gene expression vs. normalized gene read counts in *Srf* neonatal or adult KO models. Differentially expressed genes are colored red. **c,** IPA upstream regulator analysis predicted inhibitory factors in *Srf* neonatal KO data. **d**, Comparative GSEA analysis of the same GO terms in both neonatal and adult *Srf* KO models. Signed normalized enrichment scores of top down-regulated GO terms in adult *Srf* KO model were plotted.

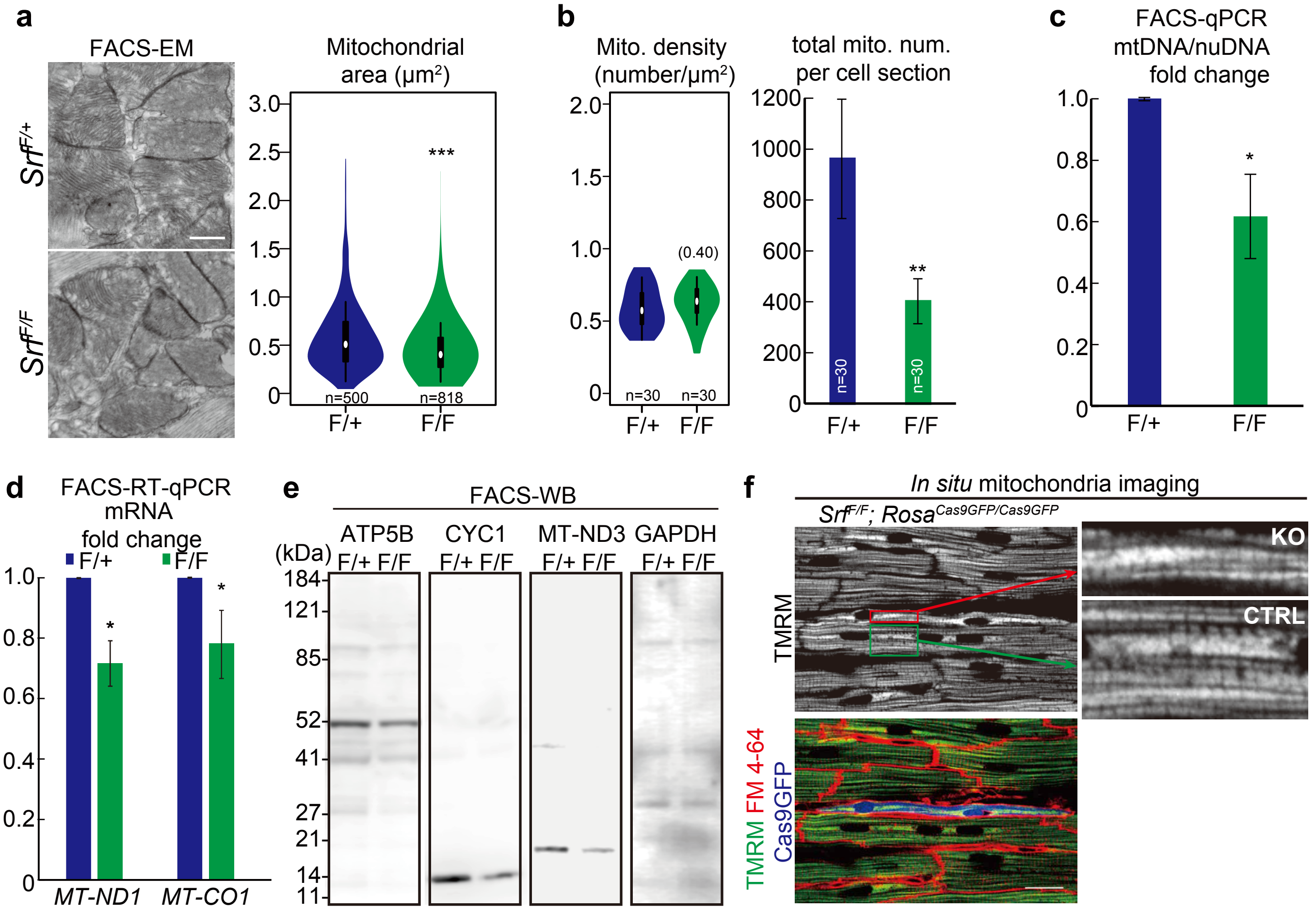

Supplementary Figure 5. *Srf* regulates mitochondrial maturation. a-b, Effect of *Srf* neonatal KO on mitochondria as assessed by FACS-EM. Representative FACS-EM images of mutant and control mitochondria are shown to the left. Scale bar, 500 nm. Indicated paramters were quantified from FACS-EM images. **c,** Effect of *Srf* neonatal KO on mitochondrial DNA copy number. qPCR analysis of mitochondrial DNA (mtDNA) to nuclear DNA (nuDNA) ratio in FACS-sorted FP+ CMs. n=4 hearts per group. **d,** RT-qPCR analysis of the expression of mitochondria-encoded genes in FACS-sorted FP+ CMs. n=4 hearts per group. **e**, Western blot analysis of mitochondrial proteins or GAPDH controls in FACS-sorted FP+ CMs. **f,** *In situ* imaging of mitochondria by TMRM labeling. Scale bar, 20 μm. Two-tailed student's *t*-test: *P<0.05, **P<0.01, ***P<0.001. Non-significant P values were labelled in parenntheses. Markings on violin plots are described in Figure 1. Bar plots indicate mean ± SD.

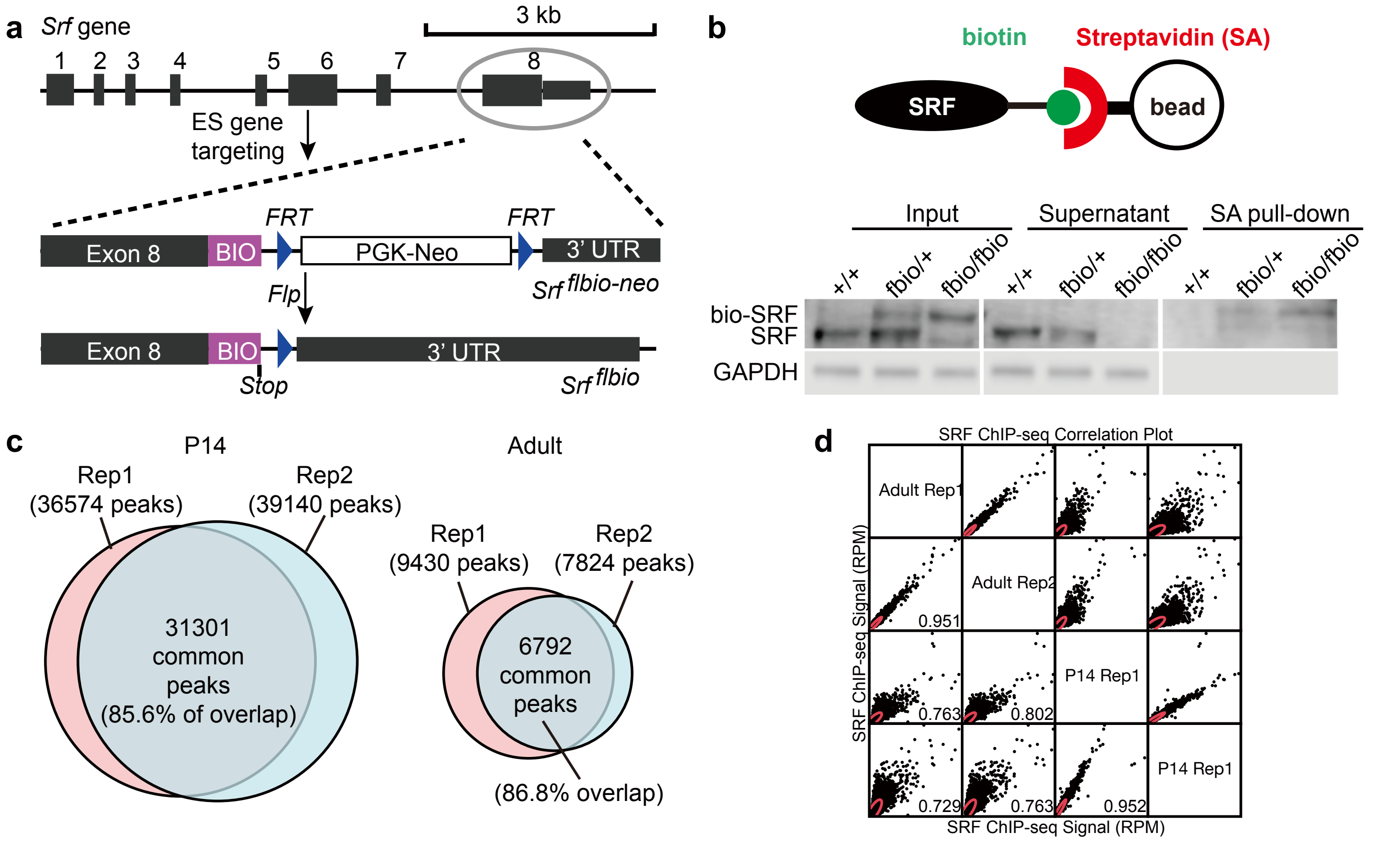

Supplementary Figure 6. BioChIP-seq analysis of developmental changes in SRF chromatin occupancy. a, Schematic of the generation of *Srf*^{fbio} knock-in mice. **b,** Western blot analysis of streptavidin (SA) pulldown validates biotin labeling of endogenous SRF protein. **c,** Biological repeats show consistent identification of SRF-bound regions at P14 and adult stages by MACS2. **d,** Pair-wise correlation of bioChIP-seq signal between individual P14 and adult data. Correlation coefficients are labeled in the plots.

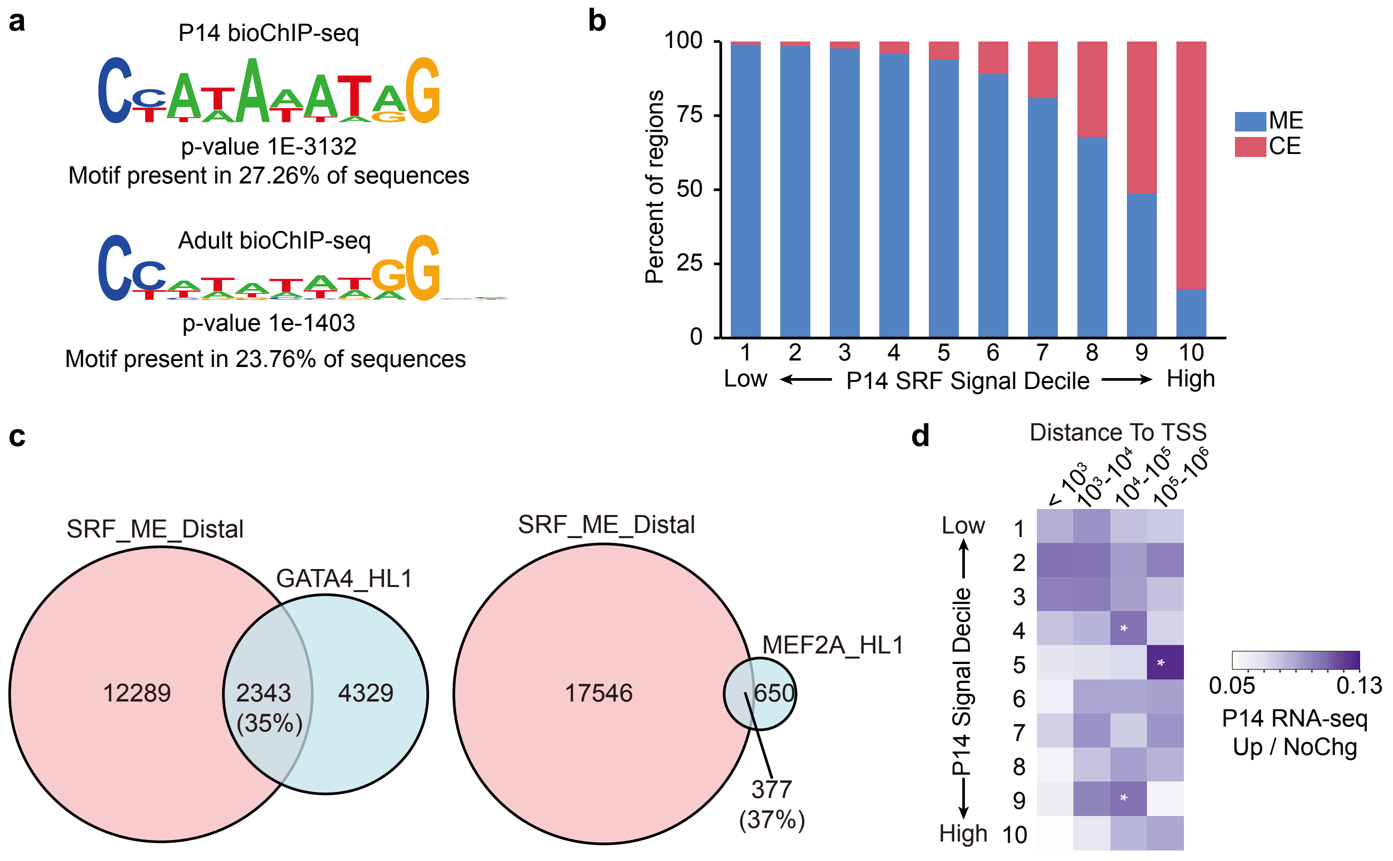

Supplementary Figure 7. BioChIP-seq analysis of developmental changes in SRF chromatin occupancy. a, *De novo* motif discovery identified the SRF binding motif as the top hit in both P14 and adult stages. **b,** Relationship between SRF occupancy signal at P14 and persistence of SRF occupancy in adult heart. ME and CE indicate regions occupied by SRF only at P14 or at P14 plus adult, respectively. **c**, Venn diagram showing overlap between distal maturation-specific Srf elements and GATA4- or MEF2A-bound regions that were previously found in HL1 cells. **d**, Relationship between SRF occupancy signal and distance to adjacent TSS with likelihood of gene upregulation. One-tailed Fisher exact test: *P<0.05.

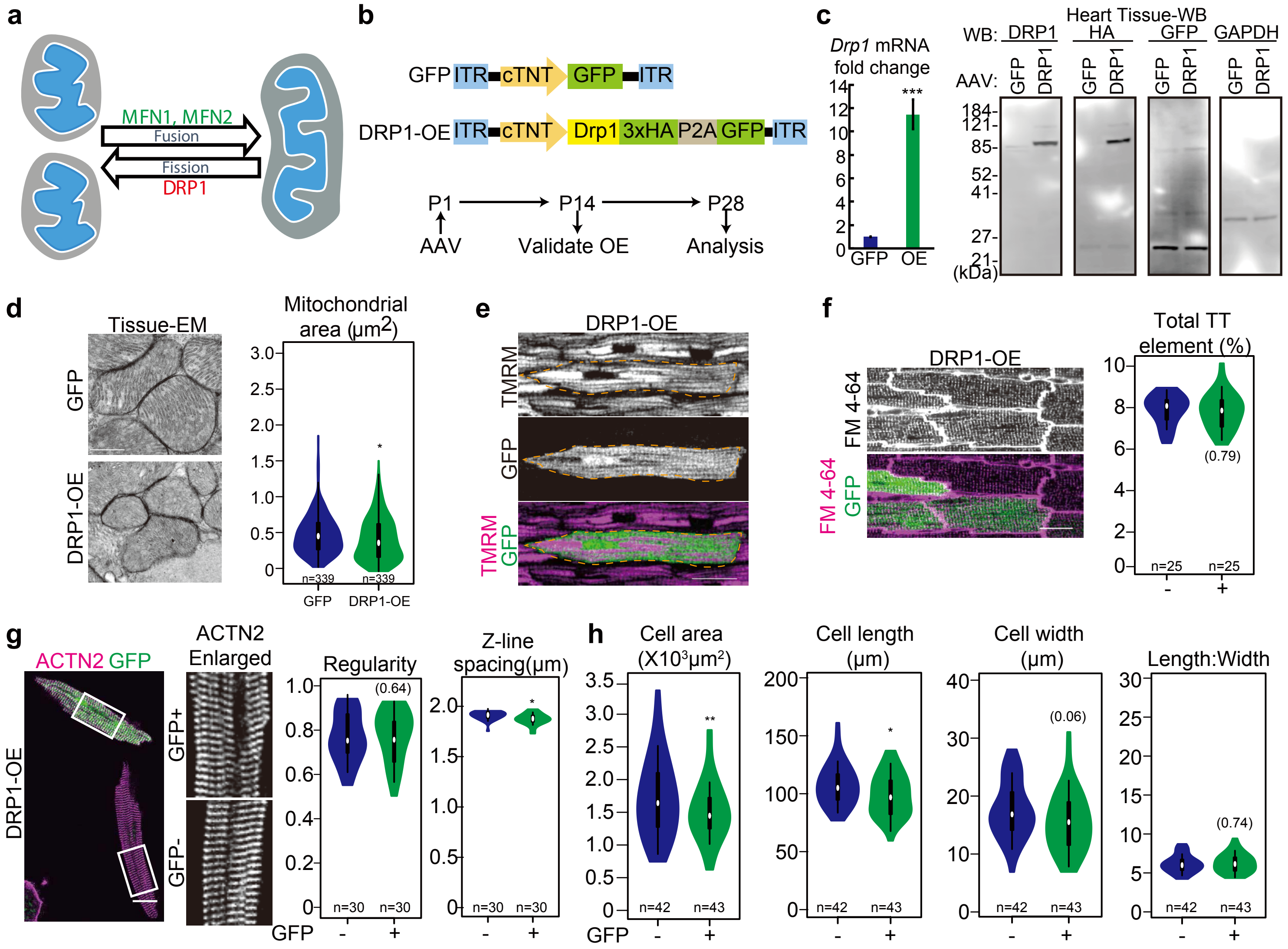

Supplementary Figure 8. *Drp1* overexpression exerts a minor impact on CM maturation. a, Schematic overview of *Drp1* as a mitochondira fission activator, counteracting the role Mfn1/2 in mitochindra dynamics. **b**, Experimental design of perinatal overexpression of DRP1. **c**, more than ten folds of DRP1 overexpression was validated by RT-qPCR (left, n = 4 hearts) and Western blot (right) in heart tissues that were infected with a high dose of AAVs (2×10^{10} vg/g). **d**, EM analysis validated decreased mitochondria size upon DRP1 overexpression in heart tissues that were infected with a high dose of AAVs. **e**, Mitochondria organization by TMRM staining and in situ imaging. **f**, In situ T-tubule imaging and AutoTT analysis revealed no T-tubule defects in DRP1-overexpressing CMs. **g-h**, a minor defects in z-line spacing (**g**) and cell size (**h**) could be detected in DRP1-over expressing CMs. Two-tailed student's t-test: *P<0.05, **P<0.01, ***P<0.001. Non-significant P values are shown in parentheses. Scale bar, 20 μm. Markings used in violin plots are described in Figure 2.

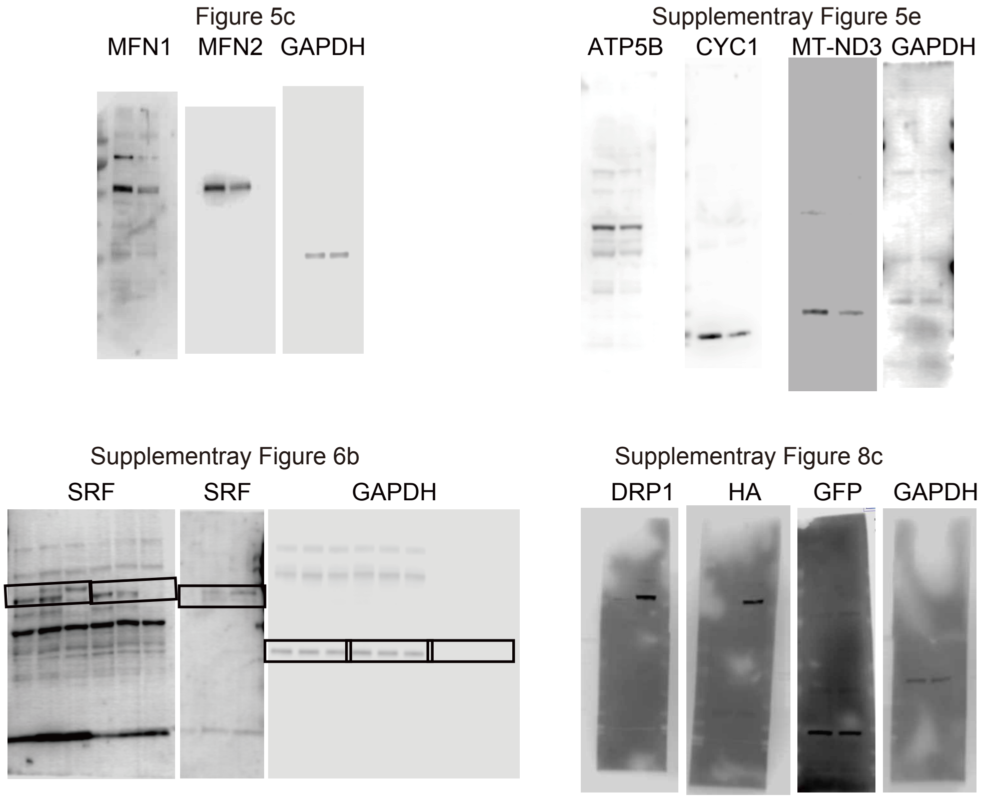

Supplementary Figure 9. Uncropped Western blot results that were presented in other figures.

Supplementary Table 1. CASAAV gRNA design

Gene	gRNA1	gRNA2	Mutagenesis validation
*Gata4**	GAGAGAGTGTGTCAATTGTG	na	Validated depletion by IF
Gata6	GCTCTATATGAAACTCCATG	na	Validated depletion by IF
*Tbx5**	TGGCCTGGCGCGCACGCCTC	CAAGTCTCCATCATCCCCGC	Validated by floxed alleles
Srf	GGGACTCGCGGGGCGAACGG	TCTTGATCTTCACGCGGCCC	Validated depletion by IF
Mef2a	TCACACGCATAATGGATGAG	ACATTTCTATGTAGCGTGGC	Not validated
*Mef2c**	ACAACGAGCCGCACGAGAGC	CCATGTCAGTGCTGGCGTAC	Not validated
*Nkx2.5**	TGGCCTCGAGGCGCGCAGAC	GACCCTCGGGCGGATAAAAA	Validated depletion by IF
*Tead1**	CCGATTGACAACGACGCGGA	TGGCTATCTATCCGCCGTGT	Validated depletion by IF
Hand2	CGGCACCGCTGGCGTACTCG	CCAACCGCAAGGAGCGGCGC	Not validated
Myh6	CAGGCACGAAGCACTCCGTG	CGCCCAGATGGCTGACTTCG	Validated depletion by IF

*The same gRNAs were also used in our previous studies[1,2] .
IF = immunofluorescent imaging

Supplementary Table 2. QPCR and RT-PCR Primers

Target	Primer 1	Primer 2	Assay
Nppa	TTCCTCGTCTTGGCCTTTTG	CCTCATCTTCTACCGGCATC	Sybr Green
Nppb	GTCCAGCAGAGACCTCAAAA	AGGCAGAGTCAGAAACTGGA	Sybr Green
MT-CO1	TGAAACCCCCAGCCATAAC	GGGTGCCCAAAGAATCAGA	Sybr Green
MT-ND1	GCCCCCTTCGACCTGACA	CGGAAGCGTGGATAAGATGC	Sybr Green
Gapdh	AGGTCGGTGTGAACGGATTTG	TGTAGACCATGTAGTTGAGGTCA	Sybr Green
AAV-titer	TCGGGATAAAAGCAGTCTGG	CCCAAGCTATTGTGTGGCCT	Sybr Green
mtDNA	CCTATCACCCTTGCCATCAT	GAGGCTGTTGCTTGTGTGAC	Sybr Green
nuDNA	TTGTCTCCTGCGACTTCAAC	GTCATACCAGGAAATGAGCTTG	Sybr Green
Myh6 deletion by CASAAV	CAGGATTCTCTGAAAAGTTAACCAG	CCCCTTCCCGGGACACGACCTTGGC	RT-PCR primers

Supplementary Table 3. Antibodies

Antigen	Host Species	Vendor	Cat. #	Working concentration	Application
SRF	Rabbit	Santa Cruz	sc-335	1:200	IF
				1:2000	WB
ACTN2	Mouse	Abcam	ab9465	1:500	IF
MYH	Mouse	DSHB	A4.1025	1:1000	IF
CAV3	Goat	Santa Cruz	sc-7665	1:250	IF
JPH2	Rabbit	Life Technologies	405300	1:250	IF
ATP5B	Mouse	Abcam	ab14730	1:2000	WB
CYC1	Rabbit	Abcam	ab90529	1:2000	WB
MT-ND3	Rabbit	Abcam	ab204977	1:2000	WB
MFN1	Mouse	Abcam	ab57602	1:2000	WB
MFN2	Rabbit	Abcam	ab124773	1:2000	WB
GAPDH	Rabbit	Santa Cruz	sc-25778	1:2000	WB
DRP1	Rabbit	Abcam	ab184247	1:2000	WB
HA	Rabbit	Abcam	ab9110	1:2000	WB

IF = immunofluorescent imaging. WB = western blotting.

Supplementary Table 4. Dyes

Name	Vendor	Cat. #	Working concentration	Application
FM 4-64	Invitrogen	T-3166	2 μg/ml	In situ imaging
TMRM	Invitrogen	T668	200 nM	In situ imaging
Fluo-4	Invitrogen	F14201	5 μM	Calcium imaging
Rhod-2	Invitrogen	R1245MP	5 μM	Calcium imaging
WGA-633	Invitrogen	W32466	5 μg/ml	Immunofluorescent imaging
DAPI	Invitrogen	D3571	500 nM	Immunofluorescent imaging

Supplementary Table 5.
Taqman probes

Target	Assay ID (Thermo Fisher)
Srf	Mm00491032_m1
Mfn1	Mm00612599_m1
Mfn2	Mm00500120_m1
Myh6	Mm00440359_m1
Myh7	Mm00600555_m1
Actc1	Mm00473657_m1
Tnni1	Mm00502426_m1
Tnni3	Mm00437164_m1
Gapdh	Mm99999915_g1
Drp1(Dnm1l)	Mm01342903_m1

Supplementary Reference

1. Guo, Y. et al. Analysis of Cardiac Myocyte Maturation Using CASAAV, A Platform for Rapid Dissection of Cardiac Myocyte Gene Function In Vivo. Circ. Res. (2017). doi:10.1161/CIRCRESAHA.116.310283

2. VanDusen, N. J., Guo, Y., Gu, W. & Pu, W. T. CASAAV: A CRISPR-Based Platform for Rapid Dissection of Gene Function In Vivo. Curr. Protoc. Mol. Biol. 120, 31.11.1–31.11.14 (2017).
