# Supplementary material for: Hierarchical and stage-specific regulation of murine cardiomyocyte maturation by serum response factor
Source: Nat Commun. 2018 Sep 21;9:3837. doi: 10.1038/s41467-018-06347-2 (PMC6155060; doi:10.1038/s41467-018-06347-2)
Supplement: Supplementary file 2 — Peer review file_new [file 41467_2018_6347_MOESM2_ESM.pdf]

Reviewers' Comments:

Reviewer #1:
Remarks to the Author:
The authors have used the recently developed CASAAV system, which allow rapid knockout of genes during the neonatal period and in mosaic fashion, to probe factors important for maturation of adult cardiomyocytes. Of 9 classic transcription factors tests, only SRF presented with a histologically grossly abnormal phenotype. The affected myocytes were found to have altered t-tubules, altered contraction and impaired sarcomeric and mitochondrial gene programs. Inactivation of sarcomeric but not mitochondrial proteins phenocopied the SRF knockout results. SRF is proposed as a master regulator of cardiomyocyte maturation. SRF has already been shown to regulate many of the sarcomeric and mitochondrial genes confirmed by this study with the CASAAV system thus it is not a surprise that its absence prevents cells from maturing. Major concerns include the proposal of SRF as a master regulator of maturation without demonstration of sufficiency, the analysis of what is and is not a mature t-tubule in SRF KO cells, validation of the lack of neonatal effect of all 8 other well characterized cardiomyocyte developmental factors, and the lack of maturation in human ES derived cardiomyocytes despite the presence of SRF in such cells. These concerns are discussed below.

1. Referral to SRF as a "master regulator" implies that SRF is both necessary and sufficient. The data indicate that SRF is a necessary protein for cardiomyocyte development/maturation. However is SRF sufficient? To satisfy this requirement, data should be provided that SRF is able to mature cardiomyocytes, either neonatal or stem cell derived.

2. SRF has already been identified to be necessary for expression of t-tubule associated channels and sarcomeric genes (e.g. PMID 15950986) as well as mitochondrial genes (e.g. PMID 11038368). These previous studies also found that SRF overexpression alone failed to rescue most genes affected by SRF KO. How can these earlier data be reconciled with the newly proposed master regulator ability of SRF?

3. The 8 classic transcriptional regulators are now found to be impotent for maturation. This is a major finding which needs validation beyond the cellular imaging of Supplemental 1c. Did knockout of the other 8 fail to affect genes important to mature cardiomyocytes?

4. SRF is in stem-cell derived cardiomyocytes (e.g. PMID 29373717). Why don't these cells have mature t-tubules?

5. The SRF- cells in mosaic neonatal heart are clearly different from their neighbors. However, given the gross morphological and contractile differences, their t-tubule spacing is not very different. T-tubules should be better imaged with high resolution and ideally with EM imaging. Furthermore, the expression of classic T-tubule proteins such as LTCC, JP2, and BIN1 must be assayed.

6. Are the authors suggesting that the order of maturation is SRF, to sarcomeric genes, to mitochondrial genes? What are the data other than a failure for selective mitochondrial gene knockout to phenocopy SRF knockout?

Reviewer #2:
Remarks to the Author:
In this contribution, Guo et al. investigate the role of SRF in regulating maturation of cardiomyocytes particularly in the neonatal period through selective chromatin occupancy. While the role of SRF in maintaining sarcomere structure, contractile apparatus, calcium handling, and

cardiac gene expression has been previous established, here the authors utilize the CASAAV system to probe the cell-autonomous aspects of SRF in maturation, avoiding organ-wide dysfunction in global knockouts. The authors show that SRF is a cell-autonomous factor required in neonatal myocytes for their structural and functional maturation. Moreover, SRF uniquely binds to numerous maturation-related targets during the neonatal period. This study provides an important advance for understanding of factors regulating myocyte maturation, which is a crucial but poorly explored area in the field. The manuscript is well-written and well-organized, and is appropriate for publication pending minor revisions suggested here.

1) In Abstract, the authors state that "these data define SRF as a master regulator of CM maturation". I would suggest using "a key upstream regulator" instead, as this study shows a permissive role of SRF rather than an instructive role in myocyte maturation.

2) In Fig s1b, the authors provide a list of candidate genes used in the initial screening. Presumably, these transcription factors were selected due to their known importance in cardiomyocyte differentiation and gene expression programs, but it would be helpful if the authors could elaborate on the selection criteria.

3) The authors state that quantification of cell size (Fig 1c) was performed following isolation, which involved size filtration of myocytes (with 100um filter) followed through flow cytometry (through a 130um nozzle). These filtrations may bias towards smaller myocytes. It is also possible that larger Srf KO myocytes are more fragile. The authors should discuss the potential size bias of the isolation procedure further (particularly with relation to gene expression measurements).

4) Fig 3 elegantly shows the unique chromatin occupancy of SRF during maturation. This argument would be strengthened if the authors could show that SRF KO (particularly in the neonatal period) eliminates the maturation element occupancy. If ChIP-seq is prohibitive, it would be illustrative to include ChIP for several critical maturation elements.

5) The authors propose a hierarchical regulatory mechanism for maturation in Fig 5h, based on comparing the phenotype of Mfn1/2 KO vs Myh6 KO. However, the data in Fig 4 do not seem to show a strong cell-autonomous regulatory role of Mfn1/2 on mitochondrial organization (as opposed to what may be organ-level effects); thus, it is difficult to draw conclusions regarding hierarchical importance of structural vs metabolic maturation. The authors would need to perform experiments analogous to Fig s4d-i for the Mfn1/2 and Myh6 KOs.

Reviewer #3:
Remarks to the Author:
In the manuscript titled “Hierarchical regulation of cardiomyocyte maturation by serum response factor,” Guo et al characterize the role of the transcription factor serum response factor (SRF) in neonatal and adult mouse cardiomyocytes and highlight its regulation of cardiomyocyte maturation. While the field of cardiac development has been successful in characterizing the regulators involved in heart development and programming of stem cells to cardiomyocytes, knowledge of signaling pathways controlling the transition of fetal to the mature adult cardiomyocyte has been limiting. Here, SRF was identified through a screen as a transcription factor important for cardiomyocyte maturation, through regulation of cardiomyocyte size, sarcomere structure and contractile function in the fetal cardiomyocyte. These features were unaffected upon knockout in the adult cardiomyocyte. These findings emphasize the importance of studying the stage-specific functions of transcription factors and regulatory pathways. The authors examine SRF regulation of gene expression through RNA-seq and ChIP-seq analyses and identify features of maturation-associated genes. In depth characterization of Mfn1/Mfn2 and Myh6 depletion, downstream targets of SRF, demonstrates a potential hierarchical role for SRF in mediating cardiomyocyte maturation phenotype.

Overall, this is an interesting paper from a good group. It provides insight into the regulation of cardiomyocyte maturation in vivo and serves as a resource for SRF-mediated cardiac gene regulation. There are some areas that should be improved, however.

Major comments:
1- The authors claim that there is hierarchical regulation of cardiomyocyte maturation mediated by SRF. This stems from the findings that Mfn1/2 depletion does not affect sarcomere organization or T-tubule content while Myh6 is upstream because it affects structural features and mitochondrial organization. The SRF data were surprising to me and advanced my thinking about maturation. On the other hand, the downstream hierarchical regulation by SRF needs further support or a revision in writing. It is not so surprising that deleting Myh6 causes failure to form myofibrils, and hence failure to have sarcomeric order to T tubules and mitochondria. It seems that any structural element of maturation that is dependent on myofibrils/sarcomeres would be disrupted by their absence. Downstream functional assays that would demonstrate the maturation phenotype endpoint, such as metabolic maturation (mitochondrial measurements as in Supplementary Figure 4), action potentials or Ca2+ handling would support a more direct relationship between structural regulation by Myh6 and functional maturation. If the authors are claiming sarcomere maturation is the driver of cardiomyocyte maturation, perhaps a rescue experiment to test whether Myh6 repletion corrects all of the effects of SRF deletion would strengthen these findings.
2- Upon Mfn depletion, there are no effects on T-tubule development, sarcomere organization or cell size/dimensions (Figure 4). Did the authors check to see if the perturbation was effective by checking for features of mitochondria biogenesis, as performed in Supplementary Figure 4?
3- Have the authors checked whether expression of GFP alone is sufficient to cause phenotypes observed with the SRF KO in Figure 1?
4- What is the rationale for using heterozygous mice (F/+) as controls (instead of F/F mice with AAV-cTnT-GFP) for Supplementary Figures 3 and 4 and Figures 2 and 3? How do the levels of SRF compare to control cells transduced with cTnT-GFP?
5- In Figure 3, authors show global reduction in ChIP signal in the adult samples. To discount any technical differences between neonatal and adult cardiomyocytes, they should show a control plot (for IgG or pulldown for another stable protein) to demonstrate similar pulldown efficiency between cell types.
6- In Figure 3E, the data suggests overlap between SRF binding and other transcription factor motifs. This leads to the conclusion that SRF interactions with other transcription factors contribute to the differences in constitutive versus maturation SRF occupancy. Is there evidence of these transcription factors showing overlapping expression or interaction with SRF? The authors have previously performed ChIP-seq for cardiac transcription factors in HL1 cells (He et al 2011). Though a different system, does the HL1 data provide support for SRF co-occupancy with other transcription factors at these identified sites?
7- What is the expression of SRF across development (neonatal vs adult cardiomyocyte)? This might provide insights and complement ChIP-seq analyses (Figure 3B).

Minor comments:
1- Please add the list of all TFs screened to the text.
2- Were the changes in expression levels of the transcription factors (Supplementary Figure 1) from the screen confirmed? Were the GFP+ cells confirmed to reduced expression since no phenotype was observed?
3- For Figure 2, it would be helpful to also show extent of change (fold change or z score) in addition to the p-value that is shown.
4- The timelines used for some of the analyses are unclear and should be noted in the figure legends (Supplementary Figure 1, Figure 5 Myh6 deletion). How does time point used for analysis of Myh6 depletion compare to Mfn1/2 depletion? What time point is shown for ChIP in Figure 3F (neonatal or adult)?
5- While the authors cite articles describing analyses of T-tubule elements and regularity, a brief description of these measurements may be helpful for the reader as these measurements are used

throughout the manuscript. Descriptions of methods used for quantifications of defective Z line and M line measurements are missing.
6- Are the signals shown in Figure 3F called-peaks?
7- Different strategies for deletion are used throughout the paper. For Figure 1, which AAV construct is used? Is it the AAV-cTnT-Cre or the AAV based on CASAAV system that is shown in Figure Supplementary 1A? The methods are unclear in text, figure and legend for the approach used in Figure 1.

Reviewer #4:
Remarks to the Author:
Guo et al. uses a strategy of mosaic deletion of SRF in the neonatal and adult cardiomyocyte to probe its cell autonomous role in cardiac maturation versus homeostasis. The authors find that SRF is essential for multiple aspects of maturation (genes controlling mitochondria remodeling, sarcomere maturation, and T-Tubule formation) but not adult CM maintenance. ChIP-Seq and RNA-seq suggest that the transition from maturation to homeostasis in associated with a reduction in the extent of SRF-bound chromatin, strength of SRF binding, and expression of maturation-specific SRF-target genes. Finally, the authors delete candidate SRF-dependent and maturation-specific genes from the mitochondria / respiration GO term (Mfn1/2) and the sarcomere GO term (Myh6). This study found that sarcomere disruption leads to mitochondria/respiration abnormalities; however, disruption of mitochondria biogenesis did not significantly impact sarcomere structure or function. This study leads to the identification of a “maturation-specific” gene program that may prove useful in devising strategies to improve maturation of iPS derived CM. The overall study is convincing and impactful; however, a few comments are provided that might strengthen the argument and improve clarity.

Comments:
1. The authors begin this study with a screen of candidate CM-transcription factors. They show that only Srf deletion impacts T-Tubule formation. However, they did not demonstrate successful deletion of the TFs that did not lead to a phenotype. This data should be included, either by qPCR or immunostaining of AAVCAS-GFP+ CM.
2. The disruption of CM maturation by Srf deletion (or Myh6 deletion) led to dramatic alterations in morphology and mitochondria disorganization. However, the TMRM staining in Supplementary Fig. 4i does not seem significantly different from other CM that did not express Cas9GFP (CM at the top and bottom of the image also seem to show alterations in the perinuclear region). Is it possible to quantify % of Cas9GFP+ CM that display mitochondria abnormalities, and/or choose a more representative image? Second, is mitochondria dysfunction in neonatal Srf KO CM associated with cell death?
3. Fig. 1 shows that myocyte structure is much more severely disrupted by Srf deletion in the neonatal period than adult. However, adult CM still display considerable alterations in CM dimensions and function. Indeed, the reduction in adult CM FS% seems to be very similar to that of neonatal. Furthermore, the authors state that contracted sarcomere length is reduced in adult Srf KO CM (Fig. 1h); however it looks to be increased in the violin plot (is this a typo in the text?). Can the authors speculate on the discrepancy between structural characteristics (much more pronounced in neonatal in Fig. 1a,b vs. 1e,f) and functional differences (present in both neonatal and adult)? RNA-seq and ChIP-seq experiments in the following figures did not seem to explain this disconnect.
4. The text states that the PCA Plot in Fig. 2b demonstrates a greater separation in the neonatal KO than in the adult; however, this is not evident in the plot that seems to show equivalent or even less separation in the neonatal KO. The authors should quantify the variance in neonatal versus adult KO shown in the PCA chart to back up this claim or alter the text. Fig. 2e and f demonstrate the top downregulated GO-terms found in neonatal Srf KO and show how these are altered in the adult KO. Surprisingly, every term that was down in the neonatal KO was upregulated in the adult KO; can the authors comment on this finding: is it significant? The

converse analysis should also be shown (top downregulated GO-terms in adult KO and how these are altered in neonatal KO).
5. Fig. 3 describes SRF chromatin occupancy that indicates cooperativity with different TFs near maturation specific (ME: Mef2 and GATA) and constitutive gene (CE: ETS, KLF) programs. It would strengthen this argument based on motif analyses if the authors could confirm using Re-ChIP that maturation-enriched interactions are lost at relevant DNA elements in the adult CM and that interactions with MEF2 and GATA are retained in relevant CE genes. Are the TFs that seem to be important for the maturation specific SRF targets lost in adult CM based on the RNA-seq? Alternatively, could they repressed or re-distributed in adult CM? Can the authors speculate on the cause of this differential interaction predicted by motif analysis?
6. TMRM changes in Fig. 4g are not apparent, and should be more convincingly shown to argue that mitochondria disorganization does not lead to autonomous changes in sarcomere structure (especially considering the changes in T-Tubules presented in Fig. 4d with possibly very minor alterations in mitochondria structure).

We thank the editors and reviewers for their constructive comments. In this revision we comprehensively addressed all the prior concerns, in many cases with new data. These changes are outlined in the point-by-point responses below.

Reviewer #1 (Remarks to the Author):

The authors have used the recently developed CASAAV system, which allow rapid knockout of genes during the neonatal period and in mosaic fashion, to probe factors important for maturation of adult cardiomyocytes. Of 9 classic transcription factors tests, only SRF presented with a histologically grossly abnormal phenotype. The affected myocytes were found to have altered t-tubules, altered contraction and impaired sarcomeric and mitochondrial gene programs. Inactivation of sarcomeric but not mitochondrial proteins phenocopied the SRF knockout results. SRF is proposed as a master regulator of cardiomyocyte maturation. SRF has already been shown to regulate many of the sarcomeric and mitochondrial genes confirmed by this study with the CASAAV system thus it is not a surprise that its absence prevents cells from maturing. Major concerns include the proposal of SRF as a master regulator of maturation without demonstration of sufficiency, the analysis of what is and is not a mature t-tubule in SRF KO cells, validation of the lack of neonatal effect of all 8 other well characterized cardiomyocyte developmental factors, and the lack of maturation in human ES derived cardiomyocytes despite the presence of SRF in such cells. These concerns are discussed below.

We appreciate these constructive comments and have provided additional data to address each of these concerns below:

1. Referral to SRF as a "master regulator" implies that SRF is both necessary and sufficient. The data indicate that SRF is a necessary protein for cardiomyocyte development/maturation. However is SRF sufficient? To satisfy this requirement, data should be provided that SRF is able to mature cardiomyocytes, either neonatal or stem cell derived.

We agree that testing sufficiency of SRF in maturation is critical, thus we performed additional experiments to test the impact of SRF overexpression on CM maturation in maturing CMs in vivo (Fig. 7 and 8). Strikingly, overexpression of SRF also perturbs the key maturation markers such as hypertrophic growth, sarcomere/mitochondria organization and T-tubule formation. RNA-seq analysis shows ectopic activation of a transcription program upon SRF overexpression that is completely distinct from the transcriptional downregulation upon SRF KO (2.2% overlap). This leads to a new insight that SRF signaling needs to be tightly balanced at an intermediate level to ensure proper maturation.

Based on this new data, we refrained from using the label “master regulator” of CM maturation. Although a master regulator could certainly show an optimal dose, above or below which the regulated process does not proceed normally, we did not want to focus on the semantics. We modified the text to call SRF an “essential regulator” of maturation instead of a “master regulator”. We appreciate this critical and constructive comment.

2. SRF has already been identified to be necessary for expression of t-tubule associated channels and sarcomeric genes (e.g. PMID 15950986) as well as mitochondrial genes (e.g. PMID 11038368). These previous studies also found that SRF overexpression alone failed to rescue most genes affected by SRF KO. How can these earlier data be reconciled with the newly proposed master regulator ability of SRF?

Our new data show that SRF activity must be properly balanced for CM maturation. This is consistent with the finding that SRF overexpression failed to rescue genes affected by SRF KO in previous studies, as is mentioned by the reviewer. Future studies are required to elucidate the mechanisms behind this interesting dosage-dependent effect of SRF.

Although the role of SRF in regulating the expression of a number of channels and sarcomere genes has been studied in the context of heart organ-wide knockouts, the general role of SRF in maturing CMs in a healthy heart (rather than in pathological conditions) has not been appreciated previously, nor has genome-wide analysis of SRF’s role in maturing CMs. Such information was indeed inaccessible previously without use of the genetic mosaic strategy and genome-wide analysis of purified CMs. This is a critical point, because pathological conditions such as heart failure themselves affect CM maturity (e.g. gene expression and T-tubules). Overcoming these difficulties is a highlight of our study that shows a maturation-specific role of SRF in a healthy heart. We added this information into discussion.

This new, rich information not uncovered in prior SRF studies in the heart leads to the following novel insights: First, despite multiple reports of cardiac SRF knockout, its broad role impacting all measured parameters of maturation has not been appreciated. This is not a general property of all cardiac TFs, as shown by our screen of 9 cardiac TFs. Second, our study showed that SRF’s critical role in regulating these aspects of maturation are limited to developing cardiomyocytes: mosaic SRF knockout in adult heart had mild effects, which is surprising given the phenotype of organ-wide adult SRF knockout (PMID: 16260633). Third, despite several other studies in which SRF was inactivated in the heart, its role in regulating mitochondrial biogenesis was not appreciated, likely due to secondary effects from organ wide knockout that obscured this signal. Although SRF regulation of mitochondria has been noted previously in other cell types (PMID: 22927399), the proposed mechanism was through regulation actin

dynamics. PMID 11038368 referenced by the reviewer is an in vitro study analyzing the regulation of the CPT1 promoter. There is not data in it that would suggest that SRF has an key role in regulating mitochondrial dynamics. Our study found direct SRF binding to endogenous mitochondria genes and regulation of their expression in vivo, which thereby directly affected mitochondrial dynamics. Fourth, although many papers appreciate the role of SRF in regulating sarcomere genes, our data show that SRF regulates myofibrillar isoform switching, which is a unique process of CM maturation that has not been shown in other studies. Furthermore, we show that sarcomeres are critical for CM maturation.

3. The 8 classic transcriptional regulators are now found to be impotent for maturation. This is a major finding which needs validation beyond the cellular imaging of Supplemental 1c. Did knockout of the other 8 fail to affect genes important to mature cardiomyocytes?

We agree we do not have enough data to support the idea that the other 8 TFs are not required for any aspect of maturation. Such validation will require doing almost every assay in this paper for each of the 8 TFs, which would be nearly equivalent to another 8 papers. However, T-tubule formation is a hallmark of CM maturation, and CASAAV directed at the other 8 TFs did not disrupt T-tubulation, suggesting that these factors are not globally required for CM maturation. CASAAV-based depletion of Gata4, Tead1, and Nkx2-5 were validated in our previous publication (PMID: 28356340 and PMID: 28967995). In the revised manuscript, we provided new data to validate Gata6 and Tbx5 results (Supplementary Fig. 1). With all these data, we believe Srf harbors unique properties that are distinct to other 8 factors in CM maturation.

The goal of describing the TF screen is to show how we arrived at SRF for further study. The focus of this paper is on SRF and not the other factors and we do not intend to rule out the role of other 8 TFs in all aspects of maturation. We revised the text to make this point more clear. Indeed, some of the negative findings of the 8 TFs are likely due to redundancy between TFs. For example, Gata4 and Gata6 are known to be redundant in CM maturation (PMID:26023924). The four isoforms of Mef2 (Mef2a, Mef2b, Mef2c and Mef2d) are also likely to perform redundant functions in CM maturation, although additional experiments are needed to test this using compound mutants.

4. SRF is in stem-cell derived cardiomyocytes (e.g. PMID 29373717). Why don't these cells have mature t-tubules?

Our new SRF overexpression data showed that SRF is not sufficient for T-tubule formation. Overexpression of SRF is indeed detrimental to T-tubule formation. Additionally, SRF activity is strongly regulated by interaction with ELK TFs, which are regulated by phosphorylation, and MRTF-family coactivators, which are regulated by

actin dynamics. It is possible that stem-cell derived CMs need additional factors, such as hormones and nuclear receptor signaling (PMID: 28974554), to induce T-tubulation. Finally, stem-cell derived CMs exhibit sarcomere disarray in unpatterned monolayer culture, which we show is incompatible with multiple aspects of CM maturation. We added this point into the discussion of the revised manuscript.

5. The SRF- cells in mosaic neonatal heart are clearly different from their neighbors. However, given the gross morphological and contractile differences, their t-tubule spacing is not very different. T-tubules should be better imaged with high resolution and ideally with EM imaging. Furthermore, the expression of classic T-tubule proteins such as LTCC, JP2, and BIN1 must be assayed.

The T-tubule defects are dramatic and can be immediately recognized by visually inspecting micrographs. However, to enhance objectivity, we used AutoTT, a well-established software to objectively and quantitatively analyzed T-tubule content in SRF- cells. This software normalized T-tubule content to the gross cell morphology, therefore circumventing the impact of cell morphology changes on T-tubule analysis (PMID: 24940790).

EM imaging of T-tubules is not currently feasible for the mosaic knockout, because the standard EM fixative is not compatible with FACS sorting of FP+ CMs. We are able to FACS sort PFA-fixed cells and perform more limited EM that provides gross information (eg. crude mitochondria and myofiber organization) but we are not confident about T-tubule observation using this method.

In the revised paper we provided additional immunostaining data of CAV3 and JPH2, two well-established T-tubule associated proteins to further validate T-tubule defects in cell-autonomous SRF knockout (Supplementary Fig. 2a).

6. Are the authors suggesting that the order of maturation is SRF, to sarcomeric genes, to mitochondrial genes?

No. Both sarcomeric and mitochondrial genes are direct downstream targets of SRF, which are supported by our RNA-seq and ChIP-seq data. The hierarchical regulation model emphasizes dependence of some maturation phenotypes on one set of maturation phenotypes but not others, i.e. T-tubulation depends on sarcomere organization but not mitochondrial biogenesis. Mitochondrial organization also depends on sarcomere organization. These observations create a hierarchy in which sarcomere organization is essential for multiple other morphological features of maturation, but mitochondrial biogenesis is not.

What are the data other than a failure for selective mitochondrial gene knockout to phenocopy SRF knockout?

Here we show that postnatal MFN1/2KO did not impact other parameters of CM maturation. In new data added to the revision, we show that the knockout of MFN1/2 did

alter mitochondrial morphology, which confirmed that mitochondria morphology did not impact other aspect of morphological maturation.

To further support the conclusion that impairing mitochondrial dynamics does not affect CM growth, T-tubulation, or sarcomere organization, in the revised paper we provide new data over expressing Drp1 in maturing CMs. This treatment causes decreased mitochondria size, but there are only very mild sarcomere or T-tubule defects. This new data is in agreement with a recent paper, which shows that mitochondrial fragmentation does not affect baseline heart development and function (PMID: 29107503).

Furthermore, in addition to these data presented in the revised paper, we recently carefully characterized another critical mitochondrial factor TFAM. TFAM depletion disrupts both morphology and function of mitochondria, but is not able to phenocopy the disruption of CM maturity observed in SRF knockout (PMID:29021295).

With all these data, we strongly believe mitochondria performs a minor role in CM maturation in the first few weeks after birth. We believe the discrepancy to prior studies (such as prior Mfn1/2 studies) is either due to confounding secondary effects of heart dysfunction, or due to a different time window of gene knockout.

Reviewer #2 (Remarks to the Author):

In this contribution, Guo et al. investigate the role of SRF in regulating maturation of cardiomyocytes particularly in the neonatal period through selective chromatin occupancy. While the role of SRF in maintaining sarcomere structure, contractile apparatus, calcium handling, and cardiac gene expression has been previous established, here the authors utilize the CASAAV system to probe the cell-autonomous aspects of SRF in maturation, avoiding organ-wide dysfunction in global knockouts. The authors show that SRF is a cell-autonomous factor required in neonatal myocytes for their structural and functional maturation. Moreover, SRF uniquely binds to numerous maturation-related targets during the neonatal period. This study provides an important advance for understanding of factors regulating myocyte maturation, which is a crucial but poorly explored area in the field. The manuscript is well-written and well-organized, and is appropriate for publication pending minor revisions
suggested here.

We appreciate the positive comments made by this reviewer.

1) In Abstract, the authors state that "these data define SRF as a master regulator of CM maturation". I would suggest using "a key upstream regulator" instead, as this study shows a permissive role of SRF rather than an instructive role in myocyte maturation.

We performed additional experiments and confirmed that SRF activity must be carefully titrated to achieve normal CM maturation. Overexpression of SRF also perturbed CM maturation (Fig. 7-8). We revised the abstract and text to reflect the new data.

2) In Fig. S1b, the authors provide a list of candidate genes used in the initial screening. Presumably, these transcription factors were selected due to their known importance in cardiomyocyte differentiation and gene expression programs, but it would be helpful if the authors could elaborate on the selection criteria.

We revised the text per this suggestion.

3) The authors state that quantification of cell size (Fig 1c) was performed following isolation, which involved size filtration of myocytes (with 100 um filter) followed through flow cytometry (through a 130um nozzle). These filtrations may bias towards smaller myocytes. It is also possible that larger Srf KO myocytes are more fragile. The authors should discuss the potential size bias of the isolation procedure further (particularly with relation to gene expression measurements).

Flow cytometry is not used in cell size quantification. Instead the cells are briefly cultured to allow them to adhere to the culture dish before measuring them under a microscope. The advantage of quantifying isolated CMs using this method is that we can position every cell in the same orientation and we can accurately measure cell length and width.

We agree that heart dissociation could potentially affect quantification of cell size. Thus in the revised manuscript, we added measurement of cell size on tissue sections to confirm the cell size defects (Supplementary Fig. 2b).

4) Fig 3 elegantly shows the unique chromatin occupancy of SRF during maturation. This argument would be strengthened if the authors could show that SRF KO (particularly in the neonatal period) eliminates the maturation element occupancy. If ChIP-seq is prohibitive, it would be illustrative to include ChIP for several critical maturation elements.

We performed bioChIP-seq of endogenous biotin-tagged SRF which is a highly sensitive and selective method that circumvents the concerns of antibody specificity and affinity issues in regular ChIP experiments. The superb performance of this method has been validated in a couple of our previous publications (PMID:28121289; PMID: 21415370), thus we are highly confident that our chromatin occupancy data is valid. It is not possible to simultaneously knockout a factor and immunoprecipitate it. We apologize if we have misconstrued the suggested experiment.

5) The authors propose a hierarchical regulatory mechanism for maturation in Fig 5h, based on comparing the phenotype of Mfn1/2 KO vs Myh6 KO. However, the data in Fig 4 do not seem to show a strong cell-autonomous regulatory role of Mfn1/2 on mitochondrial organization (as opposed to what may be organ-level effects); thus, it is difficult to draw conclusions regarding hierarchical importance of structural vs metabolic maturation. The authors would need to perform experiments analogous to Fig s4d-i for the Mfn1/2 and Myh6 KOs.

We appreciate this concern that Mfn1/2KO does not show strong cell-autonomous mitochondria defects, which was also surprising to us. To verify the mitochondrial defects upon MfnKO, we did additional EM analysis of MFNKO cells. We found a decrease of mitochondria size in MFNKO cells, which validated a mitochondrial fusion defect in these cells (Fig. 5d).

To further address the potential limitations of the mosaic MFN1/2 KO model, we decided to take a complementary approach by overexpressing DRP1, a factor that activates mitochondria fission (Supplementary Fig. 8) DRP1 overexpression caused mitochondrial fragmentation, but grossly normal T-tubule and sarcomere organization was retained in this context. These data are indeed consistent with a recent paper, which shows that mitochondrial fragmentation does not affect baseline heart development and function (PMID: 29107503).

Furthermore, in addition to these data presented in the revised paper, we recently carefully characterized another critical mitochondrial factor TFAM. TFAM depletion disrupts both morphology and function of mitochondria, but is not able to phenocopy the disruption of CM maturity observed in SRF knockout (PMID:29021295).

With all these data, we strongly believe mitochondria performs a minor role in CM maturation at least in the first few weeks after birth. We believe the discrepancy to prior studies (such as prior Mfn1/2 studies) is either due to confounding secondary effects of heart dysfunction in prior studies, or to an earlier “window of vulnerability” of maturation to Mfn1/2 (e.g. during fetal development).. The extent of MFN depletion and kinetics is at least as rapid and severe as observed in SRF KO. Thus we can at least conclude that the defects in mitochondrial dynamics as a consequence of SRF KO are not sufficient to directly impact the other parameters of maturation that occur in the SRF KO CMs.

--

Reviewer #3 (Remarks to the Author):

In the manuscript titled “Hierarchical regulation of cardiomyocyte maturation by serum response factor,” Guo et al characterize the role of the transcription factor serum response factor (SRF) in neonatal and adult mouse cardiomyocytes and highlight its regulation of cardiomyocyte maturation. While the field of cardiac development has

been successful in characterizing the regulators involved in heart development and programming of stem cells to cardiomyocytes, knowledge of signaling pathways controlling the transition of fetal to the mature adult cardiomyocyte has been limiting. Here, SRF was identified through a screen as a transcription factor important for cardiomyocyte maturation, through regulation of cardiomyocyte size, sarcomere structure and contractile function in the fetal cardiomyocyte. These features were unaffected upon knockout in the adult cardiomyocyte. These findings emphasize the importance of studying the stage-specific functions of transcription factors and regulatory pathways. The authors examine SRF regulation of gene expression through RNA-seq and ChIP-seq analyses and identify features of maturation-associated genes. In depth characterization of Mfn1/Mfn2 and Myh6 depletion, downstream targets of SRF, demonstrates a potential hierarchical role for SRF in mediating cardiomyocyte maturation phenotype.

Overall, this is an interesting paper from a good group. It provides insight into the regulation of cardiomyocyte maturation in vivo and serves as a resource for SRF-mediated cardiac gene regulation. There are some areas that should be improved, however.

We appreciate these comments.

Major comments:
1- The authors claim that there is hierarchical regulation of cardiomyocyte maturation mediated by SRF. This stems from the findings that Mfn1/2 depletion does not affect sarcomere organization or T-tubule content while Myh6 is upstream because it affects structural features and mitochondrial organization. The SRF data were surprising to me and advanced my thinking about maturation. On the other hand, the downstream hierarchical regulation by SRF needs further support or a revision in writing. It is not so surprising that deleting Myh6 causes failure to form myofibrils, and hence failure to have sarcomeric order to T tubules and mitochondria. It seems that any structural element of maturation that is dependent on myofibrils/sarcomeres would be disrupted by their absence. Downstream functional assays that would demonstrate the maturation phenotype endpoint, such as metabolic maturation (mitochondrial measurements as in Supplementary Figure 4), action potentials or Ca2+ handling
would support a more direct relationship between structural regulation by Myh6 and functional maturation. If the authors are claiming sarcomere maturation is the driver of cardiomyocyte maturation, perhaps a rescue experiment to test whether Myh6 repletion corrects all of the effects of SRF deletion would strengthen these findings.

We appreciate the perspective that it is not so surprising to see organization of T-tubule and mitochondria to be dependent on Myh6 and myofibrils. However, to our best

knowledge, thanks to the power of mosaic knockout analysis our study is the first to provide direct evidence supporting this causal relationship within the context of a normally functioning heart.

In this study we already showed clearly that structural defects contribute to functional defects. For example, in SRF KO cells, T-tubule defects partially contribute to Ca handling defects. Sarcomere defects partially contribute to contractility defects. In the case of Myh6KO, it is almost certain that functional assays like contractility and Ca2+ will show defects because of the lack of sarcomere and T-tubule. Metabolic assay is technically difficult because under mosaic KO, it is very difficult to acquire enough cells and to retain their metabolic state of cells through isolation and purification.

SRF regulates many other sarcomere genes, such as actin, troponin and MLCs. Replenishing Myh6 alone is unlikely to assemble sarcomeres and rescue SRF depletion. In addition Myh6 is a large protein that cannot be delivered through AAV, so that we currently do not have the tools to replenish Myh6 in the heart.

2- Upon Mfn depletion, there are no effects on T-tubule development, sarcomere organization or cell size/dimensions (Figure 4). Did the authors check to see if the perturbation was effective by checking for features of mitochondria biogenesis, as performed in Supplementary Figure 4?

We appreciate this concern that Mfn1/2KO may not show strong cell-autonomous mitochondria defects. To verify the mitochondrial defects upon Mfn1/2 KO, we did additional EM analysis of MFNKO cells. We found decrease of mitochondria size in Mfn1/2 KO cells, which is consistent with mitochondria fusion defects (Fig. 5d).

To further address the potential limitations of the mosaic Mfn1/2 KO model, we decided to take a complementary approach by overexpressing DRP1, a factor that activates mitochondria fission (Supplementary Fig. 8). DRP1 overexpression causes mitochondrial fragmentation, but grossly normal T-tubule and sarcomere organization is retained in this context. These data are consistent with a recent paper, which shows that mitochondrial fragmentation does not affect baseline heart development and function (PMID: 29107503).

Furthermore, in addition to these data presented in the revised paper, we recently carefully characterized another critical mitochondrial factor TFAM. TFAM depletion disrupts both morphology and function of mitochondria, but is not able to phenocopy the disruption of CM maturity observed in SRF knockout (PMID:29021295).

With all these data, we strongly believe mitochondria performs a minor role in CM maturation at least in the first few weeks after birth. We believe the discrepancy to prior studies (such as prior Mfn1/2 studies) is either due to confounding secondary effects of heart dysfunction in prior studies, or to an earlier “window of vulnerability” of maturation to Mfn1/2 (e.g. during fetal development).. The extent of MFN depletion and kinetics is at least as rapid and severe as observed in SRF KO. Thus we can at least conclude

that the defects in mitochondrial dynamics as a consequence of SRF KO are not sufficient to directly impact the other parameters of maturation that occur in the SRF KO CMs.

3- Have the authors checked whether expression of GFP alone is sufficient to cause phenotypes observed with the SRF KO in Figure 1?

GFP alone does not cause heart defects. AAV-GFP has been used as control in many studies (eg. PMID: 26173818; PMID:20703310; PMID:21674736) and no heart toxicity has been reported. Here we provide representative images of CMs overexpressing GFP to show that GFP does not perturb T-tubule or sarcomeres.

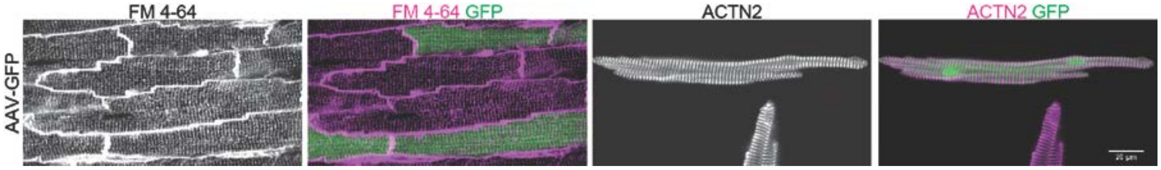

4- What is the rationale for using heterozygous mice (F/+) as controls (instead of F/F mice with AAV-cTnT-GFP) for Supplementary Figures 3 and 4 and Figures 2 and 3? How do the levels of SRF compare to control cells transduced with cTnT-GFP?

F/+ and F/F mice are littermates. F/+ with AAV-Cre is a better control than F/F with AAV-GFP for several reasons: (1) Cre + Cre-activated reporter indicates a history of Cre exposure, whereas GFP indicates active expression. These are not exactly comparable. Therefore the use of Cre + Cre-activated reporters in both groups makes the target CM populations more comparable. (2) Use of AAV-Cre in both groups better controls for both AAV batch effects. (3) Use of AAV-Cre in both groups better controls for the effects of Cre expression.

We did not observe defects in heart function or maturation in the F/+; Cre group. Although we did not observe abnormalities in F/+; Cre+ animals, if there were abnormalities they would only understate rather than overstate the abnormalities seen in F/F; Cre+.

5- In Figure 3, authors show global reduction in ChIP signal in the adult samples. To discount any technical differences between neonatal and adult cardiomyocytes, they should show a control plot (for IgG or pulldown for another stable protein) to demonstrate similar pulldown efficiency between cell types.

We performed biotin-ChIP (not antibody based ChIP), which relies on the ultra-high affinity between biotin and streptavidin. Thus protein pull-down is expected to be very efficient in both P14 and adult hearts. Indeed we recently published bioChIP-seq data of another factor and we were able to obtain robust ChIP signal using the same

method in adult heart (PMID:28121289). Thus bioChIP-seq in adult heart is a validated mature technique in our lab.

If the different Srf ChIP signals between two stages are purely due to technical issues, we would expect a proportional reduction in signal between peaks at P14 and adult, resulting in a very high correlation of ChIP signals between the two stages. However, this is not the case in our data, as the correlation coefficients between P14 and adult SRF bioChIP signals are much less than biological replicates at each stage (Supplementary Fig. 6d). Whether a P14 peak was retained or not in adult heart was not simply a reflection of the signal strength at P14 and decreased sensitivity in adult ChIP-seq. Rather it reflected the non-random change in signal strength of the peak from P14 to adult.

The globally reduced occupancy of SRF on chromatin could be biologically significant. SRF binding to chromatin is known to be modulated by its cofactors such as MRTF-A/B (PMID: 24732378). Reduced cofactor activity in adult CMs has been recently reported (PMID:29716942), which could explain the decreased SRF ChIP signal in adult CMs. Alternatively, TFs that co-occupy neonatal SRF regions could also dominate or replace SRF in adult cardiomyocytes, which would be consistent with the relatively mild role of SRF in maturity maintenance. Future studies will test these interesting hypotheses.

6- In Figure 3E, the data suggests overlap between SRF binding and other transcription factor motifs. This leads to the conclusion that SRF interactions with other transcription factors contribute to the differences in constitutive versus maturation SRF occupancy. Is there evidence of these transcription factors showing overlapping expression or interaction with SRF? The authors have previously performed ChIP-seq for cardiac transcription factors in HL1 cells (He et al 2011). Though a different system, does the HL1 data provide support for SRF co-occupancy with other transcription factors at these identified sites?

The mechanisms that direct SRF to occupy different sites during maturation is an interesting question that we are actively working on. Interaction with other TFs is likely an important mechanism. We previously mapped the binding sites of SRF, GATA4, and MEF2A (among other factors) in HL1 cells, which is a cardiomyocyte-like cell line derived from adult atrial cells. Comparing SRF occupancy data from HL1, P14, and adult, we observe that a subset of HL1 regions (3671 of 9848 regions; 37%) were also occupied by SRF in P14 and/or adult cardiomyocytes, a respectable level of agreement given the differences in technique and cellular context. To determine if SRF might co-occupy regions with GATA4 or MEF2A, as predicted by the motif analysis, we examined the HL1 data for these factors. We found SRF-bound regions in P14 hearts overlap with 35% GATA4 sites and 37% MEF2A sites in HL1 cells (Supplementary Fig. 7c) . Overall these data support SRF co-occupancy by GATA4 and MEF2A with SRF. However,

given the important technical and biological differences between systems, we are currently working on bioChIP-seq of other TFs in the heart, which will provide better answers to this question in follow-up publications.

7- What is the expression of SRF across development (neonatal vs adult cardiomyocyte)? This might provide insights and complement ChIP-seq analyses (Figure 3B).

RNA-seq data showed similar expression level of Srf between P14 and adult stage in CMs. See figure 1f (measured as RPKM).

Minor comments:

1- Please add the list of all TFs screened to the text.

We revised the manuscript per this request.

2- Were the changes in expression levels of the transcription factors (Supplementary Figure 1) from the screen confirmed? Were the GFP+ cells confirmed to reduced expression since no phenotype was observed?

CASAAV-based depletion of Gata4, Tead1, Nkx2.5 were validated in our previous publication by immunofluorescence (PMID: 28356340 and PMID: 28967995). In the revised manuscript, we provided new data to validate Gata6 and Tbx5 results (Supplementary Fig. 1). Hand2, Mef2a and Mef2c were not confirmed due to lack of antibodies effective for single cell immunofluorescent detection. However, we believe current data is sufficient to support a unique role of Srf that are distinct to other 8 factors in CM maturation.

The goal of describing the TF screen is to show how we landed on further analysis of SRF. The scope of this paper focuses on SRF but not the other factors. We do not intend to rule out the role of other 8 TFs in all aspects of maturation. We revised the text to make this point more clear.

Indeed, some of the negative findings of the 8 TFs are likely due to redundancy between TFs. For example, Gata4 and Gata6 are known to be redundant in CM maturation (PMID:26023924). The four isoforms of Mef2 (Mef2a, Mef2b, Mef2c and Mef2d) are also likely to perform redundant functions in CM maturation, although additional experiments are needed to test this using compound mutants.

3- For Figure 2, it would be helpful to also show extent of change (fold change or z score) in addition to the p-value that is shown.

We added fold change in revision per this request.

4- The timelines used for some of the analyses are unclear and should be noted in the figure legends (Supplementary Figure 1, Figure 5 Myh6 deletion). How does time point

used for analysis of Myh6 depletion compare to Mfn1/2 depletion? What time point is shown for ChIP in Figure 3F (neonatal or adult)?

Detailed timelines were added to the related figures.

Myh6 and Mfn1/2 depletion were analyzed at the same time points.

P14 bioChIP-seq signals were shown in the current Figure 4F. This information was added to the revised figure legend.

5- While the authors cite articles describing analyses of T-tubule elements and regularity, a brief description of these measurements may be helpful for the reader as these measurements are used throughout the manuscript. Descriptions of methods used for quantifications of defective Z line and M line measurements are missing.

Brief description of T-tubule measurement methods were added into the main text. Methods to measure sarcomere Z-line and M-line defects were added into the legends of Fig. 2 and Supplementary Fig. 2.

6- Are the signals shown in Figure 3F called-peaks?

Called peaks were added as bars below the ChIP signal distribution plot in that figure in revision (now in Figure 4f) .

7- Different strategies for deletion are used throughout the paper. For Figure 1, which AAV construct is used? Is it the AAV-cTnT-Cre or the AAV based on CASAAV system that is shown in Figure Supplementary 1A? The methods are unclear in text, figure and legend for the approach used in Figure 1.

Supplementary Figure 1a shows CASAAV system, which is only used for the initial screen but not for detailed analyses. Instead in Figure 2 (former Figure 1), we used AAV-cTNT-Cre to treat mice with Srf-floxed alleles. The reason is that CASAAV is convenient for screening, but Cre-mediated knockout using floxed alleles has a higher concordance between expression of fluorescent protein indicator and cells with effective protein knockout. We revised text and figures to make the methods more clear.

--

Reviewer #4 (Remarks to the Author):

Guo et al. uses a strategy of mosaic deletion of SRF in the neonatal and adult cardiomyocyte to probe its cell autonomous role in cardiac maturation versus homeostasis. The authors find that SRF is essential for multiple aspects of maturation (genes controlling mitochondria remodeling, sarcomere maturation, and T-Tubule formation) but not adult CM maintenance. ChIP-Seq and RNA-seq suggest that the transition from maturation to homeostasis in associated with a reduction in the extent of

SRF-bound chromatin, strength of SRF binding, and expression of maturation-specific SRF-target genes. Finally, the authors delete candidate SRF-dependent and maturation-specific genes from the mitochondria / respiration GO term (Mfn1/2) and the sarcomere GO term (Myh6). This study found that sarcomere disruption leads to mitochondria/respiration abnormalities; however, disruption of mitochondria biogenesis did not significantly impact sarcomere structure or function. This study leads to the identification of a “maturation-specific” gene program that may prove useful in devising strategies to improve maturation of iPS derived CM. The overall study is convincing and impactful; however, a few comments are provided that might strengthen the argument and improve clarity.

Comments:

1. The authors begin this study with a screen of candidate CM-transcription factors. They show that only Srf deletion impacts T-Tubule formation. However, they did not demonstrate successful deletion of the TFs that did not lead to a phenotype. This data should be included, either by qPCR or immunostaining of AAVCAS-GFP+ CM.

CASAAV-based depletion of Gata4, Tead1, Nkx2.5 were validated in our previous publication by immunofluorescence (PMID: 28356340 and PMID: 28967995). In the revised manuscript, we provided new data to validate Gata6 and Tbx5 results (Supplementary Fig. 1). Hand2, Mef2a and Mef2c were not confirmed due to lack of antibodies effective for single cell immunofluorescent detection. However, we believe current data is sufficient to support a unique role of Srf that are distinct to other 8 factors in CM maturation.

The goal of describing the TF screen is to show how we landed on further analysis of SRF. The scope of this paper focuses on SRF but not the other factors. We do not intend to rule out the role of other 8 TFs in all aspects of maturation. We revised the text to make this point more clear.

Indeed, some of the negative findings of the 8 TFs are likely due to redundancy between TFs. For example, Gata4 and Gata6 are known to be redundant in CM maturation (PMID:26023924). The four isoforms of Mef2 (Mef2a, Mef2b, Mef2c and Mef2d) are also likely to perform redundant functions in CM maturation, although additional experiments are needed to test this using compound mutants.

2. The disruption of CM maturation by Srf deletion (or Myh6 deletion) led to dramatic alterations in morphology and mitochondria disorganization. However, the TMRM staining in Supplementary Fig. 4i does not seem significantly different from other CM that did not express Cas9GFP (CM at the top and bottom of the image also seem to show alterations in the perinuclear region). Is it possible to quantify % of Cas9GFP+ CM

that display mitochondria abnormalities, and/or choose a more representative image? Second, is mitochondria dysfunction in neonatal Srf KO CM associated with cell death?

Although we observed decreased mitochondria size in Srf KO cells under EM, at the resolution of light microscopy, we could not detect dramatic changes in mitochondria localization and membrane potential by TMRM staining. The old Supple. Fig. 4i (new supplementary Fig. 5f) is a representative image. This is likely because SRFKO cells retained the striated organization of myofibrils. By contrast, Myh6KO completely disassembled myofibrils. Consequently the mitochondria were more disorganized in Myh6KO cells than in SrfKO cells.

We performed additional TUNEL staining but we could not detect any signal in SrfKO CMs, thus Srf depletion was unlikely to cause cell death (Supplementary Fig. 2f). Consistent with this result, we did not see apoptosis genes to be enriched in the RNA-seq data. Indeed the isolated SRF-depleted cells are healthy enough for contractility and calcium imaging assays.

3. Fig. 1 shows that myocyte structure is much more severely disrupted by Srf deletion in the neonatal period than adult. However, adult CM still display considerable alterations in CM dimensions and function. Indeed, the reduction in adult CM FS% seems to be very similar to that of neonatal. Furthermore, the authors state that contracted sarcomere length is reduced in adult Srf KO CM (Fig. 1h); however it looks to be increased in the violin plot (is this a typo in the text?). Can the authors speculate on the discrepancy between structural characteristics (much more pronounced in neonatal in Fig. 1a,b vs. 1e,f) and functional differences (present in both neonatal and adult)? RNA-seq and ChIP-seq experiments in the following figures did not seem to explain this disconnect.

Contracted sarcomere length was increased in the adult KO group. The text contained a typo that we have corrected in the revision. We apologize for the error.

Srf performs many functions in CMs by regulating a broad spectrum of genes. Some Srf functions, such as sarcomere organization and T-tubule formation, are more prominent in neonatal CMs. Other functions could be similar at different stages. For example, the roles of Srf in regulating some key Ca handling genes such as Ryr2, Slc8a1 and Atp2a2 are very similar between neonatal and adult stages (Fig 3h). Ca handling is essential for contraction/fraction shortening. This explains why we saw a contraction/fraction shortening defects in adult KO group even without structural defects as was observed in neonates.

4. The text states that the PCA Plot in Fig. 2b demonstrates a greater separation in the neonatal KO than in the adult; however, this is not evident in the plot that seems to show equivalent or even less separation in the neonatal KO. The authors should quantify the variance in neonatal versus adult KO shown in the PCA chart to back up

this claim or alter the text. Fig. 2e and f demonstrate the top downregulated GO-terms found in neonatal Srf KO and show how these are altered in the adult KO. Surprisingly, every term that was down in the neonatal KO was upregulated in the adult KO; can the authors comment on this finding: is it significant? The converse analysis should also be shown (top downregulated GO-terms in adult KO and how these are altered in neonatal KO).

Fig 2b (new Fig. 3b) shows clear separation between control and KO in neonatal KO group (labeled in round circle) but almost no separation in adult KO group (labeled in triangle). We revised the figure so that the labeling is more clear. When PCA is done only on the adult KO and control, then separation between these two groups is evident (Supplementary Fig. 4a).

Fig 2e and f (new Fig. 3e and f) show a maturation-specific role of SRF in regulating metabolism and mitochondria genes. Fig. 2f (new Fig. 3f) plots p-value and shows clearly that the differential expression of these gene are not significant (P>0.05) at adult stages. As the reviewer indicates, by GSEA analysis most of the metabolic/mitochondrial terms that were enriched among down-regulated genes in neonatal KO were enriched among up-regulated genes in the adult KO (9 of 10 had enrichment score ≤ 10-2; Fig. 2e = new Fig. 3e), consistent with a stage-specific function of SRF.

The converse analysis has been added to the revised manuscript in Supplementary Fig. 4d. This data showed heart development and muscle cell differentiation as top down-regulated GO terms in adult Srf KO model, which is consistent with previous knowledge of Srf in the heart. Of these, 4 of 10 were significantly enriched (enrichment score ≤ 10-2) in upregulated genes in neonatal KO. These data further demonstrate the unique maturation-specific role of Srf in transcriptional regulation.

5. Fig. 3 describes SRF chromatin occupancy that indicates cooperativity with different TFs near maturation specific (ME: Mef2 and GATA) and constitutive gene (CE: ETS, KLF) programs. It would strengthen this argument based on motif analyses if the authors could confirm using Re-ChIP that maturation-enriched interactions are lost at relevant DNA elements in the adult CM and that interactions with MEF2 and GATA are retained in relevant CE genes. Are the TFs that seem to be important for the maturation specific SRF targets lost in adult CM based on the RNA-seq? Alternatively, could they repressed or re-distributed in adult CM? Can the authors speculate on the cause of this differential interaction predicted by motif analysis?

The mechanisms that direct SRF to occupy different sites during maturation is an interesting question that we are actively working on. Interaction with other TFs is likely an important mechanism. We previously mapped the binding sites of SRF, GATA4, and MEF2A (among other factors) in HL1 cells, which is a cardiomyocyte-like cell line

derived from adult atrial cells. Comparing SRF occupancy data from HL1, P14, and adult, we observe that a subset of HL1 regions (3671 of 9848 regions; 37%) were also occupied by SRF in P14 and/or adult cardiomyocytes, a respectable level of agreement given the differences in technique and cellular context. To determine if SRF might co-occupy regions with GATA4 or MEF2A, as predicted by the motif analysis, we examined the HL1 data for these factors. We found SRF-bound regions in P14 hearts overlap with 35% GATA4 sites and 37% MEF2A sites in HL1 cells (Supplementary Fig. 7c) . Overall these data support SRF co-occupancy by GATA4 and MEF2A with SRF. However, given the important technical and biological differences between systems, we are currently working on bioChIP-seq of these and other TFs in the heart, which will provide better answers to this question in follow-up publications.

Re-ChIP analysis is an interesting experiment, but this is also technically challenging. In Re-ChIP, we have to use antibody to pull down at least one of the two TFs that we want to test co-occupancy, which cannot take advantage of the power of bioChIP. Heart tissue is often challenging for antibody ChIP. Currently we do not have Gata4 or Mef2 antibodies that work well for ChIP in heart tissues -- the previously validated antibodies were discontinued by Santa Cruz. Since the focus of this paper is on SRF regulation of maturation rather than TF cooperativity, we think it is appropriate to address this point in future work.

6. TMRM changes in Fig. 4g are not apparent, and should be more convincingly shown to argue that mitochondria disorganization does not lead to autonomous changes in sarcomere structure (especially considering the changes in T-Tubules presented in Fig. 4d with possibly very minor alterations in mitochondria structure).

We observed that SRF regulates genes that control mitochondrial dynamics, so we tested the hypothesis that the effect of SRF knockout was mediated by these changes. To achieve this goal, we performed mosaic Mfn1/2KO. We were also surprised to find that this did not markedly perturb mitochondrial membrane potential or CM maturation. To further examine the single cell mitochondrial defects caused by Mfn1/2 KO, we did additional EM analysis of Mfn1/2 KO cells. We found decrease of mitochondria size in Mfn1/2 KO cells, which is consistent with defective mitochondria fusion (Fig. 5d).

To further address the potential limitations of the mosaic Mfn1/2 KO model, we decided to take a complementary approach by overexpressing DRP1, a factor that activates mitochondria fission (Supplementary Fig. 8). DRP1 overexpression causes mitochondrial fragmentation, but grossly normal T-tubule and sarcomere organization is retained in this context. This data is indeed consistent with a recent paper, which shows that mitochondrial fragmentation does not affect sarcomere organization, baseline heart development and function (PMID: 29107503).

Furthermore, in addition to these data presented in the revised paper, we recently carefully characterized another critical mitochondrial factor TFAM. TFAM depletion disrupts both morphology and function of mitochondria, but is not able to phenocopy the disruption of CM maturity observed in SRF knockout (PMID:29021295).

With all these data, we strongly believe mitochondria performs a minor role in CM maturation at least in the first few weeks after birth. We believe the discrepancy to prior studies (such as prior Mfn1/2 studies) is either due to confounding secondary effects of heart dysfunction in prior studies, or to an earlier “window of vulnerability” of maturation to Mfn1/2 (e.g. during fetal development). The extent of MFN depletion and kinetics is at least as rapid and severe as observed in SRF KO. Thus we can at least conclude that the defects in mitochondrial dynamics as a consequence of SRF KO are not sufficient to directly impact the other parameters of maturation that occur in the SRF KO CMs.

Reviewers' Comments:

Reviewer #1:
Remarks to the Author:
The additional experiments and changes to the manuscript are appropriate.

Reviewer #2:
Remarks to the Author:
The authors have satisfactorily addressed my concerns. This study is expected to make significant contributions toward understanding cardiomyocyte maturation.

Reviewer #3:
Remarks to the Author:
In the revised manuscript, Guo et al have addressed our comments and performed additional analyses to strengthen their findings. Overall, this paper introduces new concepts important to the cardiac field. (1) The authors demonstrate a stage-specific role for SRF, which is required for maturation but not maintenance of cardiomyocyte phenotype. They illustrate the stage-specific loss-of-function cellular phenotypes, gene expression and SRF chromatin occupancy profiles in neonatal and adult mouse hearts. Furthermore, they performed additional overexpression experiments to show that SRF is necessary but not sufficient for driving cardiomyocyte maturation. (2) The mosaic approach for gene disruption highlights a new approach to dissect the in vivo roles for genes—in this manuscript—separating out the organ-level heart failure phenotype with cellular effects. (3) The paper introduces a novel role for SRF in metabolic maturation, specific to the neonatal system but not evident in the adult cardiomyocyte.

While the authors have incorporated major edits, I would suggest further clarification to the text regarding claims of hierarchical contribution of sarcomere organization to cardiomyocyte maturation. It is clear that SRF impact different aspects of maturation (sarcomeres, T-tubule structure, mitochondria) but whether these are distinct events branching from SRF or a through a linear sequence of dependent events is yet to be tested (Lines 387-392 and Figure 9). It should be clarified that it is not the sarcomere directly regulating mitochondrial maturation but rather sarcomere maturation holds a greater weight during maturation, more so than simply modulating metabolic maturation; or, that there is a hierarchical relationship on dependency of sarcomere structure versus mitochondrial dynamics. There is not enough evidence to indicate that sarcomere organization directly regulates cardiomyocyte maturation; this would require a rescue experiment (with Myh6) and is currently implied by Figure 9 and Discussion section.

This is an interesting paper that I likely will cite in my own work. I offer my congratulations to the authors.

Reviewer #4:
Remarks to the Author:
The authors have satisfied previous comments with new data and alterations within the text.

We appreciate the efforts of the editor and the reviewers to improve the quality of our manuscript and make it suitable for publication in Nature Communications. Here we have made point-by-point responses to reviewers' comments:

Reviewer #3 (Remarks to the Author):

While the authors have incorporated major edits, I would suggest further clarification to the text regarding claims of hierarchical contribution of sarcomere organization to cardiomyocyte maturation. It is clear that SRF impact different aspects of maturation (sarcomeres, T-tubule structure, mitochondria) but whether these are distinct events branching from SRF or a through a linear sequence of dependent events is yet to be tested (Lines 387-392 and Figure 9). It should be clarified that it is not the sarcomere directly regulating mitochondrial maturation but rather sarcomere maturation holds a greater weight during maturation, more so than simply modulating metabolic maturation; or, that there is a hierarchical relationship on dependency of sarcomere structure versus mitochondrial dynamics. There is not enough evidence to indicate that sarcomere organization directly regulates cardiomyocyte maturation; this would require a rescue experiment (with Myh6) and is currently implied by Figure 9 and Discussion section.

We agree that it is unclear whether the impact of sarcomeres on metabolic maturation is direct or indirect. We plan to further dissect this mechanism in our future studies. We edited the Discussion to clarify this point:

"Third, the diverse maturation processes downstream of SRF appear to be orchestrated in a hierarchical manner. That sarcomere inactivation was sufficient to impair multiple facets of cardiomyocyte maturation suggests that myofibrillar maturation is a dominant and essential process, and that sarcomeres are core organizers of other aspects of CM maturation. However, our data do not exclude additional direct roles of SRF in other aspects of maturation, such as mitochondrial maturation. Overall, this report provides direct demonstration of an essential and central role of sarcomeres in organizing the diverse programs of CM maturation."
